# Hyers Ulam stability and bifurcation control of leptospirosis disease dynamics and preventations: Modeling with singular and non-singular kernels

**DOI:** 10.1371/journal.pone.0314095

**Published:** 2025-03-31

**Authors:** Muhammad Farman, Aqeel Ahmad, Usama Atta, Kottakkaran Sooppy Nisar, Abdul Ghaffar

**Affiliations:** 1 Department of Computer Science and Mathematics, Lebanese American University, Beirut, Lebanon; 2 Faculty of Arts and Science, Department of Mathematics, Near East University, Nicosia North Cyprus, Turkey; 3 Department of Mathematics, Ghazi University, D. G. Khan, Dera Ghazi Khan, Pakistan; 4 Mathematics Research Center, Near East University, Nicosia North Cyprus, Turkey; 5 Department of Mathematics, College of Science and Humanities, Prince Sattam bin Abdulaziz University, Al Kharj, Saudi Arabia; Air University, PAKISTAN

## Abstract

Due to its various uses, the dynamical system is a significant research area in the field of mathematical biology. The model is first developed by applying the usual derivative with combined recovery measures of humans as well as animals for leptospirosis transmission and then converted into a generalized form of the fractal fractional sense with power law kernel, exponential law kernel, and Mittag-Leffler kernel. We verify all the fundamental characteristics of the newly developed model for the validation analysis of the system such as equilibrium points, local stability, positivity of solutions, reproductive number, and existence of a unique solution. Also, bifurcation analysis has been used for newly developed systems to observe the impact of each sub-compartment with the effect of different parameters. The results on Hyers Ulam stability are established by utilizing different kernels to observe its stable state. We used a numerical scheme based on the Lagrange polynomials for all three cases of fractal fractional derivatives having different kernels. The efficiency of the fractional operators with comparative analysis of different kernels is shown in simulation form to verify the validity and real behavior of leptospirosis transmission for humans as well as animals. he graphical explanation of our model’s solution depicts the effectiveness of our techniques applied and this study helps for future predictions and developing better control strategies.

## 1 Introduction

The world’s disease transmission patterns have been studied through the years using mathematics. Leptospirosis, Hantavirus, and Dengue are just a few of the many diseases that are prevalent in this globe [1] . Noninfectious diseases are defined as those that are typically caused by environmental or genetic factors, while infectious diseases are defined as those that are passed from person to person [2]. Animal bacterial diseases like leptospirosis can infect humans and potentially result in death. Bacterial (Leptospira interrogans) infection is major the cause of deaths. Pee from animals can contaminate water and soil, as leptospira are transferred through animal pee [3]. The Leptospira carrying animals are not injured in any way. People who have cuts on their skin (or through the mucous membranes of their mouth, nose, and eyes) and come into touch with food, drink, or soil contaminated with urine may contract the disease. Leptospirosis epidemics generally occur when people are exposed to tainted water, such as flooding [4]. Transmission from one person to another is extremely uncommon. Generally speaking, mice, raccoons, rats, sheep, goats, cattle, pigs, and dogs are the sources of bacteria [5].

These days, understanding the mechanisms underlying disease transmission and spread requires the use of mathematical modeling. Estimating the intensity and possible extent of epidemics, explaining important facets of the disease transmission process, forecasting the consequences of diseases on populations, and proposing effective control and preventative techniques have all been done with its help [6,7]. In instance, mathematical models are implemented to analyze the dynamics of infectious diseases in humans as well as animals by dividing the total population into various stages and making assumptions about the type and rate of contact from one compartment to the other. For the study of animal and human population diseases, a lot of models have been proposed [8–11].

The author analyzes stability, basic reproduction number, and sensitivity to control methods in [12] before presenting a SIR model for co-infection with dengue and Leptospirosis. Reducing contact rates and raising recovery rates are two effective ways to fight co-infections and diseases. Mathematical study of the Leptospirosis disease with saturated incidence rate in [13]. A mathematical model splits the population into human and rat subpopulation in [14], with additional categories for humans, in order to investigate disease transmission. Numerical solutions are presented, parameter conditions for disease states are established, and solution behaviors are analyzed using standard dynamic modeling.The author developed a complete system in [15] to track infection dynamics and antibody exposure in animal Leptospirosis simultaneously. The model incorporates a time-delayed SEIRS (Susceptible, Exposed, Infected, Removed, Susceptible) strategy for infection propagation together with stochastic compartmental transition rates. In order to account for varying antibody levels, it also integrates a four-state antibody kinetics model. To determine Malaysia’s relative risk of Leptospirosis, the author of [16] presented a stochastic model. An SI model was presented in [17] to study Leptospirosis in humans and animals. This model recognizes that humans can get the disease through direct contact with free-roaming bacteria in the environment as well as through contact with a variety of animals acting as bacterial reservoirs, in contrast to earlier models that mainly focused on rodents as infection vectors.

A sophisticated system of nonlinear differential equations is used in the analysis of Leptospirosis, an infectious disease, in article [18]. Because the system is complex, they use Homotopy perturbation approach to approximate the solution. For validation, the outcomes from HPM are then contrasted with those from Runge-Kutta fourth-order and nonstandard finite difference approaches. The Homotopy perturbation approach is used in to find the solution of Leptospirosis model in [19] while the non-standard finite difference scheme results along with Euler and R-K Four methods is compared in [20]. The dynamics of Leptospirosis is examined by considering rodent-human interactions into account in [21]. The basic reproduction number is calculated based on the infection, birth, and environmental carrying capacities of rats using a SI-SIR model with logistic rodent population growth. They assess the sensitivity to parameters, the persistence of the disease, and suggest management strategies. For additional information on the Leptospirosis model’s analytical and numerical solution, see [22–25].

In mathematical models to comprehend viral infections, fractional derivatives have been suggested for use. These non integer models possess more degrees of freedom, making them more precise than classical models. This criteria seems to be better met by the differential equation models with fractional orders than by those with integer orders. In order to characterize the intrinsic memory and heredity properties of numerous materials and processes, this is done so that fractional derivatives and integrals may be used.

Many scholars have utilized fractional derivative modeling to study Leptospirosis. For example, to study the spread of Leptospirosis, a fractional ordered model was presented by [26] using the multi-step Laplace Adomian decomposition approach. [27] used the Adams-Bashforth-Moulton algorithm for numerical simulation and investigated a fractional-order approach to modeling the spread of Leptospirosis. Using numerical approaches, a unique positive solution of the fractional-order Leptospirosis model is presented in [28]. The authors contrasted the Fourth-order R-K for the classical derivative with the approximate solution of MGDTM and provided numerical findings to support their conclusions. A fractional epidemic model using an Atangana Baleanu derivative is developed for the Leptospirosis in [29]. The suggested fractional model containing the ABC derivative is solved numerically using the Adams-Bashforth method, which is described below. Leptospirosis dynamics are studied in Reference [30], wherein classical-global and classical-fractional operators are used. The uniqueness of the solutions is checked, and numerical simulations for both integer and fractional orders are presented.

Besides fractional derivative, the classical derivatives are also extended to fractal derivative, where the integer order derivative can be obtained if we choose fractal dimension equal to one. In the same pattern, if the considered model possess a fractal differentiation, then the fractal derivative will be $\gamma \tau^{\gamma }$. Because of the relationship between fractal and fractional calculus, a new field is developed known as fractal fractional calculus by [31]. The derivatives mentioned above have two dimensions, the first dimension represents fractional value and the second dimension is for the fractal operator. This new concept is more useful than fractal and fractional order derivatives. Because, we can investigate real life problems by utilizing both of these operators simultaneously, i.e. if we choose the fractional dimension equal to one, we can obtain the system having only fractal order, and if we select the fractal order equal to one, the obtained system will be fractional order system [32,33]. The developed operators are very useful in those sceneries when we face a complicated behavior which cannot be described using fading, crossover or a power-law memory [34].

Because of a variety of applications, a lot of researches based on the analysis of mathematical models of different real phenomenons using fractal fractional operators have been carried out in the recent years. Here, we are presenting a summary of the few studies. For example, [35] proposed the integrator circuit model by the fractal fractional operator in the sense of Mittag Leffler kernel. [36] proposed a fractal fractional model for Malaria disease using fractal derivatives with power law and exponential decay kernels with a flavour of control strategies. [37] suggested two Influenza mathematical models using fractal fractional derivative in the framework of Mittag Leffler and power law kernels with the consideration of logistic growth and incubation periods. [38] developed a tumor immune mathematical model to analyze the relationship between the immune system and cancer cells via Atangana-Baleanu fractal fractional operator, while [39] used three different fractal fractional derivatives in his study of circuit system involving chaos theory. More detail and useful results on fractal fractional calculus and its applications to modeling of infectious diseases can be seen in [40–42].

### 1.1 Research gap

Till now, according to authors best knowledge, only a few attempts have been made on the mathematical modeling of Leptospirosis in the sense of fractal fractional operators. Such as using Mittag-leffler law [43,44]. No one has done research on Leptospirosis modeling using exponential decay kernel nor using power law kernel, and combine measures of revered for human as well as animals. Also none of them has investigated the comparison of three different kernels with complete mathematical stability analysis in their research. To overcome this deficiency, we have first proposed a model containing Six classes and derived the fundamental characteristics of the model such as equilibrium points, positivity and boundedness etc. According to our knowledge, the current study is the first attempt on modeling of Leptospirosis disease by implementing three different fractal fractional operators Mittag Leffler, Power law and Exponential decay kernels, and combine measures of revered for human as well as animals for leptospirosis transmission.

### 1.2 Innovative contributions and motivation of study

The main contributions of the article are

- A new leptospirosis mathematical model has been proposed, firstly in the integer order derivative sense and then generalized to fractal fractional order in the sense of three different operators.
- All fundamental characteristics of the model have been presented such as equilibrium points, verifying the positivity of model and existence of non negative solutions, calculation of reproductive number and global stability using Lyapunov derivative.
- We have proved that model possess at least one solution which will be unique and Hyers Ulma stable in context of three different kernels.
- It has been verified that model has no flip bifurcation along with bifurcation diagrams.
- Numerical scheme based on two step Lagrange polynomials for three different kernels is provided with the graphical explanation to reveal the effectiveness of the proposed method for eliminating the spread of disease.

### 1.3 Organization

The structure of this research is as follows: in Sect 2, we have discussed the description and formulation of the model. Sect 3 involves some of the fundamental characteristics of the model such as equilibrium points, local stability of infectious free equilibrium points, reproductive number, positivity of the model. Sect 4 is devoted for the proofs of existence of a unique solution in the sense of different fractal fractional operators. In Sect 5, we have proved the Hyers Ulam stability theorems in the FFM and FFP setting and left the proof for FFE for readers. Sect 6 involves the bifurcation results under different parameters. Sect 7 contains the numerical schemes in the sense of three different kernels FFM, FFE, FFP. Also, we have provided graphical explanation to show the effectiveness of our results, while Sect 8 concludes our research.

### 1.4 Basic concepts

The current part establishes the prerequisite findings for fractal-fractional differentials and the relevant integrals from [45].

**Definition 1:** The fractal fractional derivative of a function *Φ* ( *τ* )  with the help of power law kernel is defined as


$$\begin{array}{c} {,}_{0}^{FFP}D^{\xi ,\gamma }\Phi (\tau )=\frac{1}{\Gamma (\;j-\xi )}\frac{d}{d\tau^{\gamma }}\int_{0}^{\tau }\Phi (\omega ){\left(\tau -\omega \right)}^{k-\xi -1}d\omega , \end{array}$$ (1)

where


dΦ(τ)dτγ= lim ⁡ τ1→τΦ(τ1)−Φ(τ)τ1γ−τγ,

and *j* − 1 ≤ *ξ* , *γ* ≤ *j*.

**Definition 2:** The fractal fractional derivative of a function *Φ* ( *τ* )  with Exponential decay kernel is defined by:


$$\begin{array}{c} {,}_{0}^{FFE}D^{\xi ,\gamma }\Phi (\tau )=\frac{B(\xi )}{1-\xi }\frac{d}{d\tau^{\gamma }}\int_{0}^{\tau }\Phi (\omega )\times \exp[-\frac{\xi }{1-\xi }(\tau -\omega )]d\omega , \end{array}$$ (2)

**Definition 3:** The derivative of a function *Φ* ( *τ* )  in the Mittag Leffler context with fractal parameter *ξ* and fractal parameter *γ* is stated as:


$$\begin{array}{c} {,}^{FFM}D^{\xi ,\gamma }\left(\Phi (\tau )\right)=\frac{AB(\xi )}{1-\xi }\frac{d}{d\tau^{\gamma }}\times \int_{0}^{\tau }E_{\xi }(-\frac{\xi }{1-\xi }{\left(\tau -\omega \right)}^{\xi })\Phi (\omega )d\omega , \end{array}$$ (3)

where 0 ≤ *ξ* , *γ* ≤ 1, and $AB(\xi )=1-\xi +(\frac{\xi }{\Gamma (\xi )}).$

**Definition 4:** A function *Φ* ( *τ* )  with *ξ* , *γ* as the fractional and fractal parameters respectively has the following integral in the sense of power law kernel is given by:


$$\begin{array}{c} {,}^{FFP}I^{\xi ,\gamma }(\Phi (\tau ))=\frac{\gamma }{\Gamma (\xi )}\int_{0}^{\tau }{\left(\tau -\omega \right)}^{\xi -1}\omega^{\gamma -1}\Phi (\omega )d\omega . \end{array}$$ (4)

**Definition 5:** A function *Φ* ( *τ* )  with *ξ* , *γ* as the fractional and fractal order respectively for the exponential kernel, we have the following integral


$$\begin{array}{c} {,}^{FFE}I^{\xi ,\gamma }(\Phi (\tau ))=\frac{\xi \gamma }{B(\xi )}\int_{0}^{\tau }\omega^{\xi -1}\Phi (\omega )d\omega +\frac{\gamma (1-\xi )\tau^{\gamma -1}\Phi (\tau )}{B(\xi )}. \end{array}$$ (5)

**Definition 6:** The *ξ* , *γ* order integral of a function *Φ* in the sense of Mittag Leffler kernel is given by:


$$\begin{array}{c} {,}^{FFM}I^{\xi ,\gamma }(\Phi (\tau ))=\frac{\xi \gamma }{AB(\xi )}\int_{0}^{\tau }\omega^{\gamma -1}{\left(\tau -\omega \right)}^{\xi -1}\Phi (\omega )d\omega +\frac{\gamma (1-\xi )\tau^{\gamma -1}\Phi (\tau )}{AB(\xi )}. \end{array}$$ (6)

**Lemma 1:** The results described below also hold for fractal fractional integrals.

1.
$${,}^{FF}I^{\xi ,\gamma }\Phi (\tau )=\frac{\gamma }{\Gamma (\xi )}\int_{0}^{\tau }\tau^{\gamma -1}{\left(\tau -\omega \right)}^{\xi -1}\Phi (\omega )d\omega .$$
2.
$${,}^{FFP}I^{\xi ,\gamma }\Phi (\tau )=\frac{1}{\Gamma (1-\xi )}\frac{d}{dt^{\gamma }}\int_{0}^{\tau }{\left(\tau -\omega \right)}^{-\xi }\Phi (\omega )d\omega .$$

## 2 Description and formulation of the model

Although Leptospirosis is a fatal disease, but early treatment and vaccination can aid in recovery of animals and humans from the infection. A lot of mathematical models have been developed to study the transmission of Leptospirosis, but a few of the works have considered the effect of several parameters on the recovery of infected animals [46–48]. In this section, we will modify the research [49] to check the impact of several parameters on the recovery of animals. For the formulation of model, we rely on the following assumptions:

1. Assumptions for humans:
  - Population for human beings is homogeneous mixing all over the time.
  - The factor of urine contaminated environment to spread Leptospirosis is ignored.
  - The infected human beings can be recovered by using some antibiotics and can also shift to the susceptible compartment again at a constant rate.
  - The death factor due to illness of Leptospirosis is not considered in the formation of model.
2. Assumptions for Animals population:
  - Only the natural death rate for animals is considered. Death rate of animals due to disease is ignored.
  - We have also assumed that infected animals can recover as there are lots of vaccines are available for them.
  - Since approximately 160 mammalian species can transmit Leptospirosis, animals are referred to all carriers of Leptospirosis.
  - Animal’s population is homogeneous mixing to each subgroup.
3. Assumptions about the relationships between humans and animals population:
  - The animal’s population cannot be infected by infected humans.
  - Only the infected animals can infect humans.

### 2.1 Variables and parameters description

We have taken into account two classes in our model: animals, and humans. Three categories are further separated for humans: susceptible, infected, and recovered. Animals are further split into Three groups: susceptible, infected animals and recovered.

$S^{\hbar }(\tau )$ denotes susceptible humans (those who can be infected in future), which can become infected by contacting with infected humans. The infected humans compartment is denoted by $I^{\hbar }(\tau )$. Some of the infected humans can be recovered at the rate of *ζ* and move the recovered class shown by ${\mathbb{R}}^{\hbar }(\tau )$. Susceptible animals group $S^{A}(\tau )$ consists of the animals with loose immunity. The susceptible animals when contact with infected animals move to the infected class $I^{A}(\tau )$. The infected animals can be recovered and move to the recovered class ${\mathbb{R}}^{A}(\tau )$. The description of the parameters used in our model is presented below.

*α*: Inflow rate of humans into the susceptible class through migration or by birth

*λ*: Natural death rate for susceptible, infected and recovered humans

*ρ*: Rate at which susceptible humans get into touch with infected animals to become infected

*δ*: The rate at which recovered humans loose immunity to move into vulnerable class

*ζ*: Rate of recovery of infected humans from disease

*ϕ*: Inflow rate of animal’s population into the susceptible class

*Λ*: Natural death rate of animal’s population

*η*: Rate at which susceptible animals get into contact with infected animals to become infected

*β*: Rate of relapse for animals

*ω*: Recovery rate of infected animals from disease

**Fig 1:**
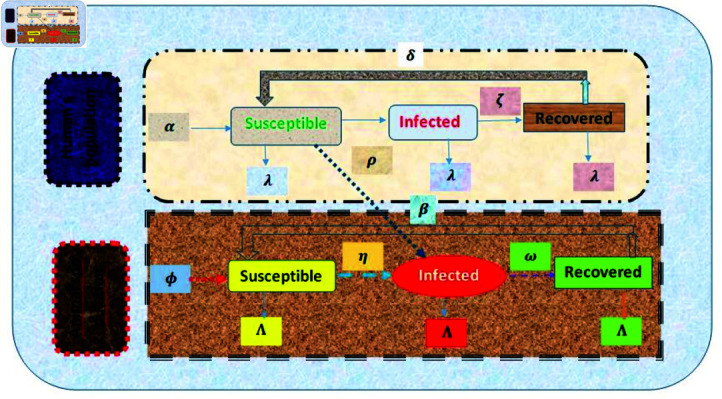
Flowchart of the newly developed Leptospirosis model.

The flow chart given below represents the relationship between the human population and animal population (Fig 1).

### 2.2 Model’s equations

Following the variables and parameters description above, we modify the Leptospirosis model suggested in [49] by inserting recovered animals term as under:


$$\begin{array}{cccc} DS^{\hbar }(\tau ) & =\alpha -(\lambda +\rho I^{A})S^{\hbar }+\delta {\mathbb{R}}^{\hbar }, & & \\ DI^{\hbar }(\tau ) & =\rho I^{A}S^{\hbar }-(\lambda +\zeta )I^{\hbar }, & & \\ D{\mathbb{R}}^{\hbar }(\tau ) & =\zeta I^{\hbar }-(\lambda +\delta ){\mathbb{R}}^{\hbar }, & & \\ DS^{A}\left(\tau \right) & =\phi -(\Lambda +\eta I^{A})S^{A}+\beta {\mathbb{R}}^{A}, & & \\ DI^{A}(\tau ) & =\eta I^{A}S^{A}-(\Lambda +\omega )I^{A}, & & \\ D{\mathbb{R}}^{A}(\tau ) & =\omega I^{A}-(\Lambda +\beta ){\mathbb{R}}^{A}. & \end{array}$$ (7)

with the initial conditions $S^{\hbar }\left(0\right)=S_{0}^{\hbar },I^{\hbar }\left(0\right)=I_{0}^{\hbar },{\mathbb{R}}^{\hbar }\left(0\right)={\mathbb{R}}_{0}^{\hbar },S^{A}\left(0\right)=S_{0}^{A},I^{A}\left(0\right)=I_{0}^{A},{\mathbb{R}}^{A}\left(0\right)={\mathbb{R}}_{0}^{A}.$

## 3 Qualitative and quantitative analysis

In this section, we will provide some essential requirements to prove the validation of our model (7) such as equilibrium points with stability, positiveness , boundedness, investigation of the invariant region, reproductive number and sensitivity of the parameters for reproductive number,

### 3.1 Equilibrium points and reproductive number

A model’s long-term behavior is highly significant. We are interested in knowing whether the system will eventually converge to a stable state after being left free for a long period of time. It is said that the system converges to a disease-free equilibrium if the disease constantly vanishes. Another possibility is that the illness continues and that class sizes eventually converge to an endemic equilibrium point, which is a single stable point. The disease free equilibrium points are where the disease vanishes. So there are no infected and recovered for both humans and animals. Therefore, we set $I^{\hbar }(\tau )={\mathbb{R}}^{\hbar }(\tau )=I^{A}(\tau )={\mathbb{R}}^{A}(\tau )=0.$ Hence solving for the susceptible compartment equations, the infectious free equilibrium points for our model (7) are


$$E^{0}=\left(\frac{\alpha }{\lambda },0,0,\frac{\phi }{\Lambda },0,0\right).$$

We can find endemic equilibrium points of our model by setting the system (7) equal to zero. Thus we have


$$\begin{array}{cccc} \alpha -(\lambda +\rho I^{A})S^{\hbar }+\delta {\mathbb{R}}^{\hbar } & =0, & & \\ \rho I^{A}S^{\hbar }-(\lambda +\zeta )I^{\hbar } & =0, & & \\ \zeta I^{\hbar }-(\lambda +\delta ){\mathbb{R}}^{\hbar } & =0, & & \\ \phi -(\Lambda +\eta I^{A})S^{A}+\beta {\mathbb{R}}^{A} & =0, & & \\ \eta I^{A}S^{A}-(\Lambda +\omega )I^{A} & =0, & & \\ \omega I^{A}-(\Lambda +\beta ){\mathbb{R}}^{A} & =0. & \end{array}$$ (8)

and then solving, we obtain the following endemic equilibrium points in the form


$$\begin{array}{cccc} {S^{\hbar }}^{⋆} & =\frac{(\alpha \eta (\delta +\lambda )\Lambda (\beta +\Lambda +\omega )(\lambda +\zeta ))}{\chi_{1}}, & & \\ {I^{\hbar }}^{⋆} & =\frac{-(\alpha (\delta +\lambda )(\beta +\Lambda )\rho (\Lambda^{2}-\eta \phi +\Lambda \omega ))}{\chi_{2}}, & & \\ {{\mathbb{R}}^{\hbar }}^{⋆} & =\frac{\left(\alpha (\beta +\Lambda )\rho (\Lambda^{2}-\eta \phi +\Lambda \omega )\zeta \right)}{\chi_{3}}, & \\ {S^{A}}^{⋆} & =\frac{\Lambda +\omega }{\eta }, & & \\ {I^{A}}^{⋆} & =\frac{-(\beta +\Lambda )(\Lambda^{2}-\eta \phi +\Lambda \omega )}{\eta \Lambda (\beta +\Lambda +\omega )}, & & \\ {{\mathbb{R}}^{A}}^{⋆} & =\frac{-\omega (\Lambda^{2}-\eta \phi +\Lambda \omega )}{\eta \Lambda (\beta +\Lambda +\omega )}, & & \end{array}$$ (9)

where


$$\begin{array}{llll} \chi_{1} & =\lambda (\beta \delta \eta \lambda \Lambda +\beta \eta \lambda^{2}\Lambda +\delta \eta \lambda \Lambda^{2}+\eta \lambda^{2}\Lambda^{2}-\beta \delta \Lambda^{2}\rho -\beta \lambda \Lambda^{2}\rho -\delta \Lambda^{3}\rho -\lambda \Lambda^{3}\rho +\beta \delta \eta \rho \phi \; & & \;\\ & \;+\beta \eta \lambda \rho \phi +\delta \eta \Lambda \rho \phi +\eta \lambda \Lambda \rho \phi +\delta \eta \lambda \Lambda \omega +\eta \lambda^{2}\Lambda \omega -\beta \delta \Lambda \rho \omega -\beta \lambda \Lambda \rho \omega -\delta \Lambda^{2}\rho \omega \; & & \;\\ & \;-\lambda \Lambda^{2}\rho \omega \beta \delta \eta \Lambda \zeta +\beta \lambda \eta \Lambda \zeta +\beta \eta \Lambda^{2}\zeta +\eta \lambda \Lambda^{2}\zeta -\beta \Lambda^{2}\rho \zeta -\lambda^{3}\rho \zeta +\beta \eta \rho \zeta \phi +\eta \Lambda \rho \zeta \phi \; & & \;\\ & \;+\delta \eta \Lambda \omega \zeta +\eta \lambda \Lambda \omega \zeta -\beta \Lambda \rho \omega \zeta -\Lambda^{2}\rho \omega \zeta ),\; & & \;\\ \chi_{2} & =\lambda (\beta \delta \eta \lambda \Lambda +\beta \eta \lambda^{2}\Lambda +\delta \eta \lambda \Lambda^{2}+\eta \lambda^{2}\Lambda^{2}-\beta \delta \Lambda^{2}\rho -\beta \lambda \Lambda^{2}\rho -\delta \Lambda^{3}\rho -\lambda \Lambda^{3}\rho +\beta \delta \eta \rho \phi \; & & \;\\ & \;+\beta \eta \lambda \rho \phi +\delta \eta \Lambda \rho \phi +\eta \lambda \Lambda \rho \phi +\delta \eta \lambda \Lambda \omega +\eta \lambda^{2}\Lambda \omega -\beta \delta \Lambda \rho \omega -\beta \lambda \Lambda \rho \omega -\delta \Lambda^{2}\rho \omega -\lambda \Lambda^{2}\rho \omega \; & & \;\\ & \;+\beta \delta \eta \Lambda \zeta +\beta \eta \lambda \Lambda \zeta +\delta \eta \Lambda^{2}\zeta +\eta \lambda \Lambda^{2}\zeta -\beta \Lambda^{2}\rho \zeta -\Lambda^{3}\rho \zeta +\beta \eta \rho \phi \zeta +\eta \Lambda \rho \phi \zeta +\delta \eta \Lambda \omega \zeta \; & & \;\\ & \;+\eta \lambda \Lambda \omega \zeta -\beta \Lambda \rho \omega \zeta -\Lambda^{2}\rho \omega \zeta ),\; & & \;\\ \chi_{3} & =\lambda (-\beta \delta \eta \lambda \Lambda -\beta \eta \lambda^{2}\Lambda -\delta \eta \lambda \Lambda^{2}-\eta \lambda^{2}\Lambda^{2}+\beta \delta \Lambda^{2}\rho +\beta \lambda \Lambda^{2}\rho +\delta \Lambda^{3}\rho +\lambda \Lambda^{3}\rho -\beta \delta \eta \rho \phi \; & & \;\\ & \;-\beta \eta \lambda \rho \phi -\delta \eta \Lambda \rho \phi -\eta \lambda \Lambda \rho \phi -\delta \eta \lambda \Lambda \omega -\eta \lambda^{2}\Lambda \omega +\beta \delta \Lambda \rho \omega +\beta \lambda \Lambda \rho \omega +\delta \Lambda^{2}\rho \omega +\lambda \Lambda^{2}\rho \omega \; & & \;\\ & \;-\beta \delta \eta \Lambda \zeta -\beta \eta \lambda \Lambda \zeta -\delta \eta \Lambda^{2}\zeta -\eta \lambda \Lambda^{2}\zeta +\beta \Lambda^{2}\rho \zeta +\Lambda^{3}\rho \zeta -\beta \eta \rho \phi \zeta -\eta \Lambda \rho \phi \zeta -\delta \eta \Lambda \omega \zeta \; & & \;\\ & \;-\eta \lambda \Lambda \omega \zeta +\beta \Lambda \rho \omega \zeta +\Lambda^{2}\rho \omega \zeta ).\; & & \; \end{array}$$

The basic reproductive number for our model (7) using next generation techniques [50–52] is given as:


$$R_{0}=\frac{\eta \phi }{\Lambda (\Lambda +\omega )}.$$

### 3.2 Local stability of infectious free equilibrium points

An equilibrium points possess stability if the system remains very close to that equilibrium point. That will result in the convergence to the equilibrium state. Equivalently, the minor changes will not effect on the convergence. In this part, we will establish the local stability of disease free equilibrium points.

**Theorem 1:** $E^{0}$ is locally stable if all the eigen values satisfied the condition $|arg(\kappa_{j})|>\frac{\xi \pi }{2}$, where $j\in {\mathbb{Z}}^{+}$.

**Proof:** To find the eigen values, we solve the jacobian of our model


$$J(E)=\left[\begin{array}{cccccc} -(\lambda +\rho I^{A}) & 0 & \delta & 0 & -\rho S^{\hbar } & 0\\ \rho I^{A} & -(\lambda +\zeta ) & 0 & 0 & \rho S^{\hbar } & 0\\ 0 & \zeta & -(\lambda +\delta ) & 0 & 0 & 0\\ 0 & 0 & 0 & -(\Lambda +\eta I^{A}) & -\eta S^{A} & \beta \\ 0 & 0 & \eta I^{A} & \eta S^{A}-(\Lambda +\omega ) & 0\\ 0 & 0 & 0 & 0 & \omega & -(\Lambda +\beta ) \end{array}\right],$$

Incorporating disease free equilibrium points and evaluating the determinant given below, we obtain $|J(E^{0})-\kappa I|=$


$$\left|\begin{array}{cccccc} -\lambda -\kappa & 0 & \delta & 0 & -\rho \frac{\alpha }{\lambda } & 0\\ 0 & -(\lambda +\zeta )-\kappa & 0 & 0 & \rho \frac{\alpha }{\lambda } & 0\\ 0 & \zeta & -(\lambda +\delta )-\kappa & 0 & 0 & 0\\ 0 & 0 & 0 & -\Lambda -\kappa & -\eta \frac{\phi }{\Lambda } & \beta \\ 0 & 0 & 0 & 0 & \eta \frac{\phi }{\Lambda }-(\Lambda +\omega )-\kappa & 0\\ 0 & 0 & 0 & 0 & \omega & -(\Lambda +\beta )-\kappa \end{array}\right|=0.$$

Evaluating the above determinant, we obtain the following eigen values, $\kappa_{1}=-\lambda ,\kappa_{2}=-\delta -\lambda$, $\kappa_{3}=-\Lambda ,\kappa_{4}=-\beta -\Lambda ,\kappa_{5}=-\frac{\Lambda^{2}-\eta \phi +\Lambda \omega }{\Lambda },\kappa_{6}=-\lambda -\zeta .$ Since all eigen values are negative, therefore, our system is locally asymptotic stable.

### 3.3 Positivity of the model

In this subsection, we present the explanation that the solutions are better because they illustrate real-world problems with positive values.

**Theorem 2:** The proposed model along with initial conditions is unique and is limited in ${\mathbb{R}}_{+}^{6}$ in all cases.

**Proof:**


$$\begin{array}{llll} {,}^{FF}D^{\xi ,\gamma }S^{\hbar }(\tau ) & =\alpha +\delta {\mathbb{R}}^{\hbar }\geq 0,\; & & \;\\ {,}^{FF}D^{\xi ,\gamma }I^{\hbar }(\tau ) & =\rho I^{A}S^{\hbar }\geq 0,\; & & \;\\ {,}^{FF}D^{\xi ,\gamma }{\mathbb{R}}^{\hbar }(\tau ) & =\zeta I^{\hbar }\geq 0,\; & & \;\\ {,}^{FF}D^{\xi ,\gamma }S^{A}\left(\tau \right) & =\phi +\beta {\mathbb{R}}^{A}\geq 0,\; & & \;\\ {,}^{FF}D^{\xi ,\gamma }I^{A}(\tau ) & =\eta I^{A}S^{A}\geq 0,\; & & \;\\ {,}^{FF}D^{\xi ,\gamma }{\mathbb{R}}^{A}(\tau ) & =\omega I^{A}\geq 0.\; & & \; \end{array}$$

### 3.4 Non negativity of solutions with different kernels

The part assures the positivity of the solution with non negative initial conditions.

**Theorem 3:** If the initial conditions $\left\{S^{\hbar }(0),I^{\hbar }(0),{\mathbb{R}}^{\hbar }(0),S^{A}(0),I^{A}(0),{\mathbb{R}}^{A}(0)\right\}$ are all non - negative and the solution of the model (7) exists, then the solution always remains non - negative.

**Proof:** We take start of our proof from integer order case. For this, we define the norm ∥ρ∥= sup ⁡ τ∈Dρ, where $D_{\rho }$ *stands* *for* *the* *domain* *of* *ρ*.

Our model’s (7) first equation is


DSℏ(τ)=α−(λ+ρIA)Sℏ+δℝℏ,∀ ⁡τ>0,≥− (λ+ρsup ⁡ τ∈DIA|IA| )Sℏ,∀ ⁡τ>0,=− (λ+ρ∥IA∥∞ )Sℏ,∀ ⁡τ>0,Sℏ(τ)≥S0ℏ(τ)e− (λ+ρ∥IA∥∞ )τ,∀ ⁡τ>0.

Similarly, for other equations of the model can be derived with same pattern.

Now, we prove the same results for fractal fractional case. The proof for the Mittag-Leffler case starts with the susceptible humans compartment:


⁣0FFMDξ,γSℏ (τ)=α−(λ+ρIA)Sℏ+δℝℏ,∀ ⁡τ≥0,≥−(λ+ρ|IA|)Sℏ,∀ ⁡τ≥0,≥− (λ+ρsup ⁡ τ∈DIA|IA|)Sℏ,∀ ⁡τ≥0,≥− (λ+ρ∥IA∥∞)Sℏ,∀ ⁡τ≥0.

This results in the following expression


$$S^{\hbar }(\tau )\geq S^{\hbar }(0)E_{\xi }[-\frac{c^{1-\gamma }\xi \left(\lambda +\rho ∥I^{A}{∥}_{\infty }\right)\tau^{\xi }}{AB(\xi )-(1-\xi )\left(\lambda +\rho ∥I^{A}{∥}_{\infty }\right)}],\forall \tau \geq 0,$$

where *c* is time component. The non-negativity of Mittag-Leffler kernel and initial conditions imply the non-negativity of $S^{\hbar }$.


$$\begin{array}{llll} {,}_{0}^{FFM}D^{\xi ,\gamma }I^{\hbar }\left(\tau \right) & =\rho I^{A}S^{\hbar }-(\lambda +\zeta )I^{\hbar },\forall \tau \geq 0\; & & \;\\ & \geq -(\lambda +\zeta )I^{\hbar },\forall \tau \geq 0\; & & \;\\ I^{\hbar }(\tau ) & \geq I^{\hbar }(0)E_{\xi }[-\frac{c^{1-\gamma }\xi (\lambda +\zeta )\tau^{\xi }}{AB(\xi )-(1-\xi )(\lambda +\zeta )}],\forall \tau \geq 0\; & & \; \end{array}$$

where *c* is time component. The non-negativity of Mittag-Leffler kernel and initial conditions imply the non-negativity of $I^{\hbar }$.


$$\begin{array}{llll} {,}_{0}^{FFM}D^{\xi ,\gamma }{\mathbb{R}}^{\hbar }\left(\tau \right) & =\zeta I^{\hbar }-(\lambda +\delta ){\mathbb{R}}^{\hbar },\forall \tau \geq 0,\; & & \;\\ & \geq -(\lambda +\delta ){\mathbb{R}}^{\hbar },\forall \tau \geq 0,\; & & \;\\ {\mathbb{R}}^{\hbar }(\tau ) & \geq {\mathbb{R}}^{\hbar }(0)E_{\xi }[-\frac{c^{1-\gamma }\xi (\lambda +\delta )\tau^{\xi }}{AB(\xi )-(1-\xi )(\lambda +\delta )}],\forall \tau \geq 0,\; & & \; \end{array}$$

where *c* is time component. The non-negativity of Mittag-Leffler kernel and initial conditions imply the non-negativity of ${\mathbb{R}}^{\hbar }$.


$$\begin{array}{llll} {,}_{0}^{FFM}D^{\xi ,\gamma }S^{A}\left(\tau \right) & =\phi -(\Lambda +\eta I^{A})S^{A}+\beta {\mathbb{R}}^{A},\forall \tau \geq 0,\; & & \;\\ & \geq -(\Lambda +\eta |I^{A}|)S^{A},\forall \tau \geq 0,\; & & \;\\ S^{A}(\tau ) & \geq S^{A}(0)E_{\xi }[-\frac{c^{1-\gamma }\xi (\Lambda +\eta ∥I^{A}{∥}_{\infty })\tau^{\xi }}{AB(\xi )-(1-\xi )(\Lambda +\eta ∥I^{A}{∥}_{\infty })}],\forall \tau \geq 0,\; & & \; \end{array}$$

where *c* is time component. The non-negativity of Mittag-Leffler kernel and initial conditions imply the non-negativity of $S^{A}$.


$$\begin{array}{llll} {,}_{0}^{FFM}D^{\xi ,\gamma }I^{A}\left(\tau \right) & =\eta I^{A}S^{A}-(\Lambda +\omega )I^{A},\forall \tau \geq 0,\; & & \;\\ & \geq -(\Lambda +\omega )I^{A},\forall \tau \geq 0,\; & & \;\\ I^{A}(\tau ) & \geq I^{A}(0)E_{\xi }[-\frac{c^{1-\gamma }\xi [(\Lambda +\omega )]\tau^{\xi }}{AB(\xi )-(1-\xi )[(\Lambda +\omega )]}],\forall \tau \geq 0,\; & & \; \end{array}$$

where *c* is time component. The non-negativity of Mittag-Leffler kernel and initial conditions imply the non-negativity of $I^{A}$.


$$\begin{array}{llll} {,}_{0}^{FFM}D^{\xi ,\gamma }{\mathbb{R}}^{A}\left(\tau \right) & =\omega I^{A}-(\Lambda +\beta ){\mathbb{R}}^{A},\forall \tau \geq 0,\; & & \;\\ & \geq -(\Lambda +\beta ){\mathbb{R}}^{A},\forall \tau \geq 0,\; & & \;\\ {\mathbb{R}}^{A}(\tau ) & \geq {\mathbb{R}}^{A}(0)E_{\xi }[-\frac{c^{1-\gamma }\xi (\Lambda +\beta )\tau^{\xi }}{AB(\xi )-(1-\xi )(\Lambda +\beta )}],\forall \tau \geq 0,\; & & \; \end{array}$$

where *c* is time component. The non-negativity of Mittag-Leffler kernel and initial conditions imply the non-negativity of ${\mathbb{R}}^{A}$.

Similarly, for power law kernel, we have


$$\begin{array}{llll} S^{\hbar }(\tau ) & \geq S^{\hbar }(0)E_{\xi }[-c^{1-\gamma }(\lambda +\rho ∥I^{A}{∥}_{\infty })\tau^{\xi }],\forall \tau >0,\; & & \;\\ I^{\hbar }(\tau ) & \geq I^{\hbar }(0)E_{\xi }[-c^{1-\gamma }(\lambda +\zeta )\tau^{\xi }],\forall \tau >0,\; & & \;\\ {\mathbb{R}}^{\hbar }(\tau ) & \geq {\mathbb{R}}^{\hbar }(0)E_{\xi }[-c^{1-\gamma }(\lambda +\delta )\tau^{\xi }],\forall \tau >0,\; & & \;\\ S^{A}(\tau ) & \geq S^{A}(0)E_{\xi }[-c^{1-\gamma }(\Lambda +\eta ∥I^{A}{∥}_{\infty })\tau^{\xi }],\forall \tau >0,\; & & \;\\ I^{A}(\tau ) & \geq I^{A}(0)E_{\xi }[-c^{1-\gamma }((\Lambda +\omega )-\eta ∥S^{A}{∥}_{\infty })\tau^{\xi }],\forall \tau >0,\; & & \;\\ {\mathbb{R}}^{A}(\tau ) & \geq {\mathbb{R}}^{A}(0)E_{\xi }[-c^{1-\gamma }(\Lambda +\beta )\tau^{\xi }],\forall \tau >0.\; & & \; \end{array}$$

For exponential kernel, we have


$$\begin{array}{cccc} S^{\hbar }(\tau ) & \geq S^{\hbar }(0)\exp[-\frac{c^{1-\gamma }\xi (\lambda +\rho ∥I^{A}{∥}_{\infty })\tau }{M(\xi )-(1-\xi )(\lambda +\rho ∥I^{A}{∥}_{\infty })}],\forall \tau >0, & & \\ I^{\hbar }(\tau ) & \geq I^{\hbar }(0)\exp[-\frac{c^{1-\gamma }\xi (\lambda +\zeta )\tau }{M(\xi )-(1-\xi )(\lambda +\zeta )}],\forall \tau >0, & & \\ {\mathbb{R}}^{\hbar }(\tau ) & \geq {\mathbb{R}}^{\hbar }(0)\exp[-\frac{c^{1-\gamma }\xi (\lambda +\delta )\tau }{M(\xi )-(1-\xi )(\lambda +\delta )}],\forall \tau >0, & & \\ S^{A}(\tau ) & \geq S^{A}(0)\exp[-\frac{c^{1-\gamma }\xi (\Lambda +\eta ∥I^{A}{∥}_{\infty })\tau }{M(\xi )-(1-\xi )(\Lambda +\eta ∥I^{A}{∥}_{\infty })}],\forall \tau >0, & & \\ I^{A}(\tau ) & \geq I^{A}(0)\exp[-\frac{c^{1-\gamma }\xi ((\Lambda +\omega )-\eta ∥S^{A}{∥}_{\infty })\tau }{M(\xi )-(1-\xi )(\lambda +\rho ∥I^{A}{∥}_{\infty })}],\forall \tau >0, & & \\ {\mathbb{R}}^{A}(\tau ) & \geq {\mathbb{R}}^{A}(0)\exp[-\frac{c^{1-\gamma }\xi (\Lambda +\beta )\tau }{M(\xi )-(1-\xi )(\Lambda +\beta )}],\forall \tau >0. & & \end{array}$$

### 3.5 Global stability analysis

This part discusses global stability by considering Lyapunov function and Lasalle’s principle. Linear stability provides information us about the behaviour of a system near an equilibrium point, but it does not offer insights into what happens farther away from equilibrium. The Lyapunov function can be used to determine the stability of an equilibrium point near and farther away from the equilibrium point.

**Theorem 4:** If the reproductive number $R_{0}$ exceeds 1, then the equilibrium points of our model attain global asymptotic stability.

**Proof:** Let’s define the Lyapunov function in the following manner


L (Sℏ⋆,Iℏ⋆,ℝℏ⋆,SA⋆,IA⋆,ℝA⋆)= (Sℏ−Sℏ⋆−Sℏ⋆ ln ⁡ Sℏ⋆Sℏ)+ (Iℏ−Iℏ⋆−Iℏ⋆ ln ⁡ Iℏ⋆Iℏ)+ (ℝℏ−ℝℏ⋆−ℝℏ⋆ ln ⁡ ℝℏ⋆ℝℏ)+ (SA−SA⋆−SA⋆ ln ⁡ SA⋆SA)+ (IA−IA⋆−IA⋆ ln ⁡ IA⋆IA)+ (ℝA−ℝA⋆−ℝA⋆ ln ⁡ ℝA⋆ℝA).

Taking derivative on both sides, we obtain


$$\begin{array}{llll} {,}^{FF}D^{\xi ,\gamma }L & ={\left(\frac{S^{\hbar }-{S^{\hbar }}^{⋆}}{S^{\hbar }}\right)}^{FF}D^{\xi ,\gamma }S^{\hbar }+{\left(\frac{I^{\hbar }-{I^{\hbar }}^{⋆}}{I^{\hbar }}\right)}^{FF}D^{\xi ,\gamma }I^{\hbar }+{\left(\frac{{\mathbb{R}}^{\hbar }-{{\mathbb{R}}^{\hbar }}^{⋆}}{{\mathbb{R}}^{\hbar }}\right)}^{FF}D^{\xi ,\gamma }{\mathbb{R}}^{\hbar }+\; & & \;\\ & \;{\left(\frac{S^{A}-{S^{A}}^{⋆}}{S^{A}}\right)}^{FF}D^{\xi ,\gamma }S^{A}+{\left(\frac{I^{A}-{I^{A}}^{⋆}}{I^{A}}\right)}^{FF}D^{\xi ,\gamma }I^{A}+{\left(\frac{{\mathbb{R}}^{A}-{{\mathbb{R}}^{A}}^{⋆}}{{\mathbb{R}}^{A}}\right)}^{FF}D^{\xi ,\gamma }{\mathbb{R}}^{A}.\; & & \; \end{array}$$

Using the values of ${{,}^{FF}D^{\xi ,\gamma }S}^{\hbar }{,{,}^{FF}D^{\xi ,\gamma }I}^{\hbar }{,{,}^{FF}D^{\xi ,\gamma }\mathbb{R}}^{\hbar }{,{,}^{FF}D^{\xi ,\gamma }S}^{A}{,{,}^{FF}D^{\xi ,\gamma }I}^{A}{,}^{FF}D^{\xi ,\gamma }{\mathbb{R}}^{A}$ from our model in the previous equation, we have


$$\begin{array}{c} \begin{array}{ccc} {,}^{FF}D^{\xi ,\gamma }L & =\left(\frac{S^{\hbar }-{S^{\hbar }}^{⋆}}{S^{\hbar }}\right)\left[\alpha -(\lambda +\rho I^{A})S^{\hbar }+\delta {\mathbb{R}}^{\hbar }\right]+ & \\ & \;\left(\frac{I^{\hbar }-{I^{\hbar }}^{⋆}}{I^{\hbar }}\right)\left[\rho I^{A}S^{\hbar }-(\lambda +\zeta )I^{\hbar }\right]+\left(\frac{{\mathbb{R}}^{\hbar }-{{\mathbb{R}}^{\hbar }}^{⋆}}{{\mathbb{R}}^{\hbar }}\right)\left[\zeta I^{\hbar }-(\lambda +\delta ){\mathbb{R}}^{\hbar }\right]\\ & \;+\left(\frac{S^{A}-{S^{A}}^{⋆}}{S^{A}}\right)\left[\phi -(\Lambda +\eta I^{A})S^{A}+\beta {\mathbb{R}}^{A}\right]+\left(\frac{I^{𝔸}-{I^{A}}^{⋆}}{I^{A}}\right)\left[\eta I^{A}S^{A}-(\Lambda +\omega )I^{A}\right]\\ & \;+\left(\frac{{\mathbb{R}}^{A}-{{\mathbb{R}}^{A}}^{⋆}}{{\mathbb{R}}^{A}}\right)\left[\omega I^{A}-(\Lambda +\beta ){\mathbb{R}}^{A}\right]. \end{array} \end{array}$$

Putting $S^{\hbar }=S^{\hbar }-{S^{\hbar }}^{⋆},I^{\hbar }=I^{\hbar }-{I^{\hbar }}^{⋆},{\mathbb{R}}^{\hbar }={\mathbb{R}}^{\hbar }-{{\mathbb{R}}^{\hbar }}^{⋆},S^{A}=S^{A}-{S^{A}}^{⋆},I^{A}=I^{A}-{I^{A}}^{⋆},{\mathbb{R}}^{A}={\mathbb{R}}^{A}-{{\mathbb{R}}^{A}}^{⋆},$ we have


$$\begin{array}{c} \begin{array}{ccc} {,}^{FF}D^{\xi ,\gamma }L & =\left(\frac{S^{\hbar }-{S^{\hbar }}^{⋆}}{S^{\hbar }}\right)\left[\alpha -\{\lambda +\rho (I^{A}-{I^{A}}^{⋆})\}(S^{\hbar }-{S^{\hbar }}^{⋆})+\delta ({\mathbb{R}}^{\hbar }-{{\mathbb{R}}^{\hbar }}^{⋆})\right] & \\ & \;+\left(\frac{I^{\hbar }-{I^{\hbar }}^{⋆}}{I^{\hbar }}\right)\left[\rho \{(I^{A}-{I^{A}}^{⋆})(S^{\hbar }-{S^{\hbar }}^{⋆})\}-(\lambda +\zeta )(I^{\hbar }-{I^{\hbar }}^{⋆})\right]\\ & \;+\left(\frac{{\mathbb{R}}^{\hbar }-{{\mathbb{R}}^{\hbar }}^{⋆}}{{\mathbb{R}}^{\hbar }}\right)\left[\zeta (I^{\hbar }-{I^{\hbar }}^{⋆})-(\lambda +\delta )({\mathbb{R}}^{\hbar }-{\mathbb{R}}^{\hbar })\right]\\ & \;+\left(\frac{S^{A}-{S^{A}}^{⋆}}{S^{A}}\right)\left[\phi -\{\Lambda +\eta (I^{A}-{I^{A}}^{⋆})\}(S^{A}-{S^{A}}^{⋆})+\beta ({\mathbb{R}}^{A}-{{\mathbb{R}}^{A}}^{⋆})\right]\\ & \;+\left(\frac{I^{A}-{I^{A}}^{⋆}}{I^{A}}\right)\left[\eta \{(I^{A}-{I^{A}}^{⋆})(S^{A}-{S^{A}}^{⋆})\}-(\Lambda +\omega )(I^{A}-{I^{A}}^{⋆})\right]\\ & \;+\left(\frac{{\mathbb{R}}^{A}-{{\mathbb{R}}^{A}}^{⋆}}{{\mathbb{R}}^{A}}\right)\left[\omega (I^{A}-{I^{A}}^{⋆})-(\Lambda +\beta )({\mathbb{R}}^{A}-{{\mathbb{R}}^{A}}^{⋆})\right]. \end{array} \end{array}$$

After simplifying the above expression, we have which can be written as


$${,}^{FF}D^{\xi ,\gamma }L=ℵ-ℜ,$$

where


$$\begin{array}{llll} ℵ & =\alpha +\frac{\rho }{S^{\hbar }}{I^{A}}^{⋆}{\left(S^{\hbar }-{S^{\hbar }}^{⋆}\right)}^{2}+\delta {\mathbb{R}}^{\hbar }+\delta \frac{{S^{\hbar }}^{⋆}}{S^{\hbar }}{{\mathbb{R}}^{\hbar }}^{⋆}+\rho I^{A}S^{\hbar }+\rho {I^{A}}^{⋆}{S^{\hbar }}^{⋆}+\frac{{I^{\hbar }}^{⋆}}{I^{\hbar }}\rho {I^{A}}^{⋆}S^{\hbar }+\frac{{I^{\hbar }}^{⋆}}{I^{\hbar }}\rho I^{A}{S^{\hbar }}^{⋆}\; & & \;\\ & \;+\zeta I^{\hbar }+\frac{{{\mathbb{R}}^{\hbar }}^{⋆}}{{\mathbb{R}}^{\hbar }}\zeta {I^{\hbar }}^{⋆}+\phi +\beta {\mathbb{R}}^{A}+\frac{{S^{A}}^{⋆}}{S^{A}}\beta {{\mathbb{R}}^{A}}^{⋆}+\frac{\eta }{I^{A}}{\left(I^{A}-{I^{A}}^{⋆}\right)}^{2}S^{A}+\omega I^{A}+\frac{{{\mathbb{R}}^{A}}^{⋆}}{{\mathbb{R}}^{A}}\omega {I^{A}}^{⋆},\; & & \; \end{array}$$

and


$$\begin{array}{llll} ℜ & =\frac{\lambda }{S^{\hbar }}{\left(S^{\hbar }-{S^{\hbar }}^{⋆}\right)}^{2}+\frac{\rho }{S^{\hbar }}I^{A}{\left(S^{\hbar }-{S^{\hbar }}^{⋆}\right)}^{2}+\delta {{\mathbb{R}}^{\hbar }}^{⋆}+\alpha \frac{{S^{\hbar }}^{⋆}}{S^{\hbar }}+\delta \frac{{S^{\hbar }}^{⋆}}{S^{\hbar }}{\mathbb{R}}^{\hbar }+\rho {I^{A}}^{⋆}S^{\hbar }+\rho I^{A}{S^{\hbar }}^{⋆}\; & & \;\\ & \;+\frac{\left(\lambda +\zeta \right)}{I^{\hbar }}{\left(I^{\hbar }-{I^{\hbar }}^{⋆}\right)}^{2}+\frac{{I^{\hbar }}^{⋆}}{I^{\hbar }}\rho I^{A}S^{\hbar }+\frac{{I^{\hbar }}^{⋆}}{I^{\hbar }}\rho {I^{A}}^{⋆}{S^{\hbar }}^{⋆}+\zeta {I^{\hbar }}^{⋆}+\frac{{{\mathbb{R}}^{\hbar }}^{⋆}}{{\mathbb{R}}^{\hbar }}\zeta I^{\hbar }+\frac{\left(\lambda +\delta \right)}{{\mathbb{R}}^{\hbar }}{\left({\mathbb{R}}^{\hbar }-{{\mathbb{R}}^{\hbar }}^{⋆}\right)}^{2}\; & & \;\\ & \;+\frac{\Lambda }{S^{A}}{\left(S^{A}-{S^{A}}^{⋆}\right)}^{2}+\frac{\eta }{S^{A}}I^{A}{\left(S^{A}-{S^{A}}^{⋆}\right)}^{2}+\beta {{\mathbb{R}}^{A}}^{⋆}+\frac{{S^{A}}^{⋆}}{S^{A}}\phi +\frac{{S^{A}}^{⋆}}{S^{A}}\beta {\mathbb{R}}^{A}+\frac{\eta }{I^{A}}\; & & \;\\ & \;{\left(I^{A}-{I^{A}}^{⋆}\right)}^{2}{S^{A}}^{⋆}+\frac{\Lambda +\omega }{I^{A}}{\left(I^{A}-{I^{A}}^{⋆}\right)}^{2}+\omega {I^{A}}^{⋆}+\frac{\lambda +\beta }{{\mathbb{R}}^{A}}{\left({\mathbb{R}}^{A}-{{\mathbb{R}}^{A}}^{⋆}\right)}^{2}+\frac{{{\mathbb{R}}^{A}}^{⋆}}{{\mathbb{R}}^{A}}\omega I^{A}.\; & & \; \end{array}$$

If *ℵ* < *ℜ*, then ${,}^{FF}D^{\xi ,\gamma }L<0$. If ${S^{\hbar }}^{⋆}=S^{\hbar },{I^{\hbar }}^{⋆}=I^{\hbar },{{\mathbb{R}}^{\hbar }}^{⋆}={\mathbb{R}}^{\hbar },{S^{A}}^{⋆}=S^{A},{I^{A}}^{⋆}=I^{A},{{\mathbb{R}}^{A}}^{⋆}={\mathbb{R}}^{A},{\text{then}}^{FF}D^{\xi ,\gamma }L=0.$ Hence by Lasalle’s invariance principle, our model is globally stable.

## 4 Uniqueness and existence of solutions

The following inquiries may be of interest to someone:

1. For a solution to our model to exist, what requirements need to be fulfilled?
2. For the Leptospirosis model, under what circumstances is there a unique solution?

We will find the answer of these questions for FFM, FFE and FFP kernels in the following subsections:

### 4.1 Existence with unique solution for FFM

We start the existence and uniqueness by Mittag-Leffler kernel. Consider the Leptospirosis model (7) with Mittag-Leffler memory:


$$\begin{array}{cccc} {,}^{FFM}D^{\xi ,\gamma }S^{\hbar }\left(\tau \right) & =\alpha -(\lambda +\rho I^{A})S^{\hbar }+\delta {\mathbb{R}}^{\hbar }={ℷ}_{1}\left(\tau ,ℶ\right), & & \\ {,}^{FFM}D^{\xi ,\gamma }I^{\hbar }\left(\tau \right) & =\rho I^{A}S^{\hbar }-(\lambda +\zeta )I^{\hbar }={ℷ}_{2}\left(\tau ,ℶ\right), & & \\ {,}^{FFM}D^{\xi ,\gamma }{\mathbb{R}}^{\hbar }\left(\tau \right) & =\zeta I^{\hbar }-(\lambda +\delta ){\mathbb{R}}^{\hbar }={ℷ}_{3}\left(\tau ,ℶ\right), & & \\ {,}^{FFM}D^{\xi ,\gamma }S^{A}\left(\tau \right) & =\phi -(\Lambda +\eta I^{A})S^{A}+\beta {\mathbb{R}}^{A}={ℷ}_{4}\left(\tau ,ℶ\right), & \\ {,}^{FFM}D^{\xi ,\gamma }I^{A}\left(\tau \right) & =\eta I^{A}S^{A}-(\Lambda +\omega )I^{A}={ℷ}_{5}\left(\tau ,ℶ\right), & & \\ {,}^{FFM}D^{\xi ,\gamma }{\mathbb{R}}^{A}\left(\tau \right) & =\omega I^{A}-(\Lambda +\beta ){\mathbb{R}}^{A}={ℷ}_{6}\left(\tau ,ℶ\right), & & \end{array}$$ (10)

where


$$\begin{array}{c} ℶ=\left\{S^{\hbar },I^{\hbar },{\mathbb{R}}^{\hbar },S^{A},I^{A},{\mathbb{R}}^{A}\right\}. \end{array}$$ (11)

Our model (10) will possess a unique solution, if the following two conditions are satisfied given by:


$$\begin{array}{c} \begin{array}{c} 1.\;{ℷ}_{i}\left(Z_{i},\tau \right)<{𝕜}_{i}\left(1+\left|Z_{i}\right|\right),\forall i=1,2,\cdots \;,6\\ 2.\;{∥{ℷ}_{i}\left(Z_{i},\tau \right)-{ℷ}_{i}\left(Y_{i},\tau \right)∥}_{\infty }<{𝕜}_{i}{∥Z_{i}-Y_{i}∥}_{\infty }^{2},\forall i=1,2,\cdots \;,6. \end{array} \end{array}$$

Initially,


$$\begin{array}{c} \begin{array}{cc} {\left|{ℷ}_{1}\left(\tau ,{\left(S^{\hbar }\right)}_{1},I^{\hbar },{\mathbb{R}}^{\hbar },S^{A},I^{A},{\mathbb{R}}^{A}\right)\right|}^{2} & ={\left|\alpha -(\lambda +\rho I^{A})S^{\hbar }+\delta {\mathbb{R}}^{\hbar }\right|}^{2},\\ & \leq 2\left[|\alpha {|}^{2}+|\delta {\mathbb{R}}^{\hbar }{|}^{2}|+(|\lambda {|}^{2}+|\rho {|}^{2}|I^{A}{|}^{2})|S^{\hbar }{|}^{2}\right],\\ & =2(|\alpha {|}^{2}+|\delta {\mathbb{R}}^{\hbar }{|}^{2})[1+|\frac{|\lambda {|}^{2}+|\rho {|}^{2}|I^{A}{|}^{2}}{\left(|\alpha {|}^{2}+|\delta {\mathbb{R}}^{\hbar }{|}^{2}\right)}],\\ & <{𝕜}_{1}\left(1+{\left|S^{\hbar }\right|}^{2}\right). \end{array} \end{array}$$

given that


$$\begin{array}{c} \frac{|\lambda {|}^{2}+|\rho {|}^{2}|I^{A}{|}^{2}}{\left(|\alpha {|}^{2}+|\delta {\mathbb{R}}^{\hbar }{|}^{2}\right)}<1, \end{array}$$

where


$$\begin{array}{c} {𝕜}_{1}=2(|\alpha {|}^{2}+|\delta {\mathbb{R}}^{\hbar }{|}^{2}). \end{array}$$

$$\begin{array}{c} \begin{array}{cc} |{ℷ}_{2}(\tau ,{\left(S^{\hbar }\right)}_{1},I^{\hbar },{\mathbb{R}}^{\hbar },S^{A},I^{A},{\mathbb{R}}^{A}){|}^{2} & =|\rho I^{A}S^{\hbar }-(\lambda +\zeta )I^{\hbar }{|}^{2},\\ & \leq 2[|\rho I^{A}S^{\hbar }{|}^{2}+|\lambda +\zeta {|}^{2}|I^{\hbar }{|}^{2}],\\ & =2|\rho I^{A}S^{\hbar }{|}^{2}[1+\frac{|\lambda +\zeta {|}^{2}}{|\rho I^{A}S^{\hbar }{|}^{2}}|I^{\hbar }{|}^{2}],\\ & <{𝕜}_{2}(1+|I^{\hbar }{|}^{2}). \end{array} \end{array}$$

whenever


$$\begin{array}{c} \frac{|\lambda +\zeta {|}^{2}}{|\rho I^{A}S^{\hbar }{|}^{2}}<1, \end{array}$$

where


$$\begin{array}{c} {𝕜}_{2}=2|\rho I^{A}S^{\hbar }{|}^{2}. \end{array}$$

$$\begin{array}{c} \begin{array}{cc} |{ℷ}_{3}\left(\tau ,{\left(S^{\hbar }\right)}_{1},I^{\hbar },{\mathbb{R}}^{\hbar },S^{A},I^{A},{\mathbb{R}}^{A}\right){|}^{2} & ={\left|\zeta I^{\hbar }-(\lambda +\delta ){\mathbb{R}}^{\hbar }\right|}^{2},\\ & \leq 2[|\zeta I^{\hbar }{|}^{2}+|\lambda +\delta {|}^{2}|{\mathbb{R}}^{\hbar }{|}^{2}],\\ & =2|\zeta I^{\hbar }{|}^{2}[1+\frac{|\lambda +\delta {|}^{2}}{|\zeta I^{\hbar }{|}^{2}}|{\mathbb{R}}^{\hbar }{|}^{2}],\\ & <{𝕜}_{3}\left(1+{\left|{\mathbb{R}}^{\hbar }\right|}^{2}\right). \end{array} \end{array}$$

under the condition


$$\begin{array}{c} \frac{|\lambda +\delta {|}^{2}}{|\zeta I^{\hbar }{|}^{2}}<1, \end{array}$$

In the situation where


$$\begin{array}{c} {𝕜}_{3}=2|\zeta I^{\hbar }{|}^{2}. \end{array}$$

$$\begin{array}{c} \begin{array}{cc} {\left|{ℷ}_{4}\left(\tau ,{\left(S^{\hbar }\right)}_{1},I^{\hbar },{\mathbb{R}}^{\hbar },S^{A},I^{A},{\mathbb{R}}^{A}\right)\right|}^{2} & ={\left|\phi -(\Lambda +\eta I^{A})S^{A}+\beta {\mathbb{R}}^{A}\right|}^{2},\\ & \leq 2[|\phi {|}^{2}+|\beta {\mathbb{R}}^{A}{|}^{2}+|(\Lambda +\eta )I^{A}{|}^{2}|S^{A}{|}^{2}],\\ & =2(|\phi {|}^{2}+|\beta {\mathbb{R}}^{A}{|}^{2})[1+\frac{|(\Lambda +\eta )I^{A}{|}^{2}}{|\phi {|}^{2}+|\beta {\mathbb{R}}^{A}{|}^{2}}|S^{A}{|}^{2}],\\ & <{𝕜}_{4}\left(1+{\left|S^{A}\right|}^{2}\right). \end{array} \end{array}$$

assuming that


$$\begin{array}{c} \frac{|(\Lambda +\eta )I^{A}{|}^{2}}{|\phi {|}^{2}+|\beta {\mathbb{R}}^{A}{|}^{2}}<1, \end{array}$$

where


$$\begin{array}{c} {𝕜}_{4}=2(|\phi {|}^{2}+|\beta {\mathbb{R}}^{A}{|}^{2}). \end{array}$$

$$\begin{array}{c} \begin{array}{cc} {\left|{ℷ}_{5}(\tau ,{\left(S^{\hbar }\right)}_{1},I^{\hbar },{\mathbb{R}}^{\hbar },S^{A},I^{A},{\mathbb{R}}^{A})\right|}^{2} & ={\left|\eta I^{A}S^{A}-(\Lambda +\omega )I^{A}\right|}^{2},\\ & \leq 2\left[{\left|\right|}^{2}+{\left|\nu^{𝜃}\right|}^{2}{\left|I^{A}\right|}^{2}\right],\\ & =2{\left|\left(\eta^{𝜃}I^{\hbar }+{𝕜}^{𝜃}L\right)S^{A}\right|}^{2}\left[1+\frac{{\left|\nu^{𝜃}\right|}^{2}{\left|I^{A}\right|}^{2}}{{\left|\left(\eta^{𝜃}I^{\hbar }+{𝕜}^{𝜃}L\right)S^{A}\right|}^{2}}\right],\\ & <{𝕜}_{6}\left(1+{\left|I^{A}\right|}^{2}\right). \end{array} \end{array}$$

under the condition


$$\begin{array}{c} \frac{{\left|\nu^{𝜃}\right|}^{2}}{{\left|\left(\eta^{𝜃}I^{\hbar }+{𝕜}^{𝜃}L\right)S^{A}\right|}^{2}}<1, \end{array}$$

where


$$\begin{array}{c} {𝕜}_{6}=2{\left|\left(\eta^{𝜃}I^{\hbar }+{𝕜}^{𝜃}L\right)S^{A}\right|}^{2}. \end{array}$$

$$\begin{array}{c} \begin{array}{cc} {\left|{ℷ}_{6}\left(\tau ,{\left(S^{\hbar }\right)}_{1},I^{\hbar },{\mathbb{R}}^{\hbar },S^{A},I^{A},{\mathbb{R}}^{A}\right)\right|}^{2} & ={\left|\omega I^{A}-(\Lambda +\beta ){\mathbb{R}}^{A}\right|}^{2},\\ & \leq 2[|\omega I^{A}{|}^{2}+|\Lambda +\beta {|}^{2}|{\mathbb{R}}^{A}{|}^{2}],\\ & =2|\omega I^{A}{|}^{2}[1+\frac{|\Lambda +\beta {|}^{2}}{|\omega I^{A}{|}^{2}}|{\mathbb{R}}^{A}{|}^{2}],\\ & <{𝕜}_{6}\left(1+{\left|{\mathbb{R}}^{A}\right|}^{2}\right). \end{array} \end{array}$$

under the condition


$$\begin{array}{c} \frac{|\Lambda +\beta {|}^{2}}{|\omega I^{A}{|}^{2}}<1, \end{array}$$

where


$$\begin{array}{c} {𝕜}_{6}=2|\omega I^{A}{|}^{2}. \end{array}$$

Hence condition 1 is verified. Now, we check the validation of the Lipschitz condition 2.


$$\begin{array}{c} \begin{array}{c} {\left|{ℷ}_{1}\left(\tau ,{\left(S^{\hbar }\right)}_{1},I^{\hbar },{\mathbb{R}}^{\hbar },S^{A},I^{A},{\mathbb{R}}^{A}\right)-{ℷ}_{1}\left(\tau ,{\left(S^{\hbar }\right)}_{2},I^{\hbar },{\mathbb{R}}^{\hbar },S^{A},I^{A},{\mathbb{R}}^{A}\right)\right|}^{2},\\ \leq {\left|\lambda +\rho I^{A}\right|}^{2}{\left|{\left(S^{\hbar }\right)}_{1}-{\left(S^{\hbar }\right)}_{2}\right|}^{2},\\ \leq {\bar{𝕜}}_{1}{∥{\left(S^{\hbar }\right)}_{1}-{\left(S^{\hbar }\right)}_{2}∥}_{\infty }^{2}. \end{array} \end{array}$$

where


$$\begin{array}{c} {\bar{𝕜}}_{1}={\left|\lambda \right|}^{2}+|\rho {|}^{2}∥I^{A}{∥}_{\infty }^{2}. \end{array}$$

If


$$\begin{array}{c} \begin{array}{c} {\left|{ℷ}_{2}\left(\tau ,S^{\hbar },{\left(I^{\hbar }\right)}_{1},{\mathbb{R}}^{\hbar },S^{A},I^{A},{\mathbb{R}}^{A}\right)-{ℷ}_{2}\left(\tau ,S^{\hbar },{\left(I^{\hbar }\right)}_{2},{\mathbb{R}}^{\hbar },S^{A},I^{A},{\mathbb{R}}^{A}\right)\right|}^{2},\\ \leq {\left(\lambda +\delta \right)}^{2}{\left|{\left(I^{\hbar }\right)}_{1}-{\left(I^{\hbar }\right)}_{2}\right|}^{2},\\ \leq {\bar{𝕜}}_{2}{∥\left({\left(I^{\hbar }\right)}_{1}-{\left(I^{\hbar }\right)}_{2}\right)∥}_{\infty }^{2}, \end{array} \end{array}$$

where


$$\begin{array}{c} {\bar{𝕜}}_{2}={\left(\lambda +\delta \right)}^{2}. \end{array}$$

If


 |ℷ3 (τ,Sℏ,Iℏ, (ℝℏ)1,SA,IA,ℝA)−ℷ3 (τ,Sℏ,Iℏ, (ℝℏ)2,SA,IA,ℝA)|2,≤ |− (λ+δ)|2 | (ℝℏ)1− (ℝℏ)2|2,≤ | (λ+δ)|2 sup ⁡ D∈ℝℏ| (ℝℏ)1− (ℝℏ)2|,≤𝕜¯3 ∥ (ℝℏ)1− (ℝℏ)2∥∞2,

where


$$\begin{array}{c} {\bar{𝕜}}_{3}={\left(\lambda +\delta \right)}^{2}. \end{array}$$

If


$$\begin{array}{c} \begin{array}{c} {\left|{ℷ}_{4}\left(\tau ,S^{\hbar },I^{\hbar },{\mathbb{R}}^{\hbar },{\left(S^{A}\right)}_{1},I^{A},{\mathbb{R}}^{A}\right)-{ℷ}_{4}\left(\tau ,S^{\hbar },I^{\hbar },{\mathbb{R}}^{\hbar },{\left(S^{A}\right)}_{2},I^{A},{\mathbb{R}}^{A}\right)\right|}^{2},\\ \leq \left[|\Lambda {|}^{2}+|\eta {|}^{2}|I^{A}{|}^{2}\right]{\left|{\left(S^{A}\right)}_{1}-{\left(S^{A}\right)}_{2}\right|}^{2},\\ \leq {\bar{𝕜}}_{4}{∥{\left(S^{A}\right)}_{1}-{\left(S^{A}\right)}_{2}∥}_{\infty }^{2}, \end{array} \end{array}$$

where


$$\begin{array}{c} {\bar{𝕜}}_{5}=|\Lambda {|}^{2}+|\eta {|}^{2}∥I^{A}{∥}_{\infty }^{2}. \end{array}$$

If


$$\begin{array}{c} \begin{array}{c} {\left|{ℷ}_{5}\left(\tau ,S^{\hbar },I^{\hbar },{\mathbb{R}}^{\hbar },S^{A},{\left(I^{A}\right)}_{1},{\mathbb{R}}^{A}\right)-{ℷ}_{5}\left(\tau ,S^{\hbar },I^{\hbar },{\mathbb{R}}^{\hbar },S^{A},{\left(I^{A}\right)}_{2},{\mathbb{R}}^{A}\right)\right|}^{2},\\ \leq \left[|\eta {|}^{2}|S^{A}{|}^{2}+|\Lambda +\omega {|}^{2}\right]{\left|{\left(I^{A}\right)}_{1}-{\left(I^{A}\right)}_{2}\right|}^{2},\\ \leq {\bar{𝕜}}_{5}{∥{\left(I^{A}\right)}_{1}-{\left(I^{A}\right)}_{2}∥}_{\infty }^{2}, \end{array} \end{array}$$

where


$$\begin{array}{c} {\bar{𝕜}}_{5}=|\eta {|}^{2}∥S^{A}{∥}_{\infty }^{2}+|\Lambda +\omega {|}^{2}. \end{array}$$

If


$$\begin{array}{c} \begin{array}{ccc} & {\left|{ℷ}_{6}\left(\tau ,S^{\hbar },I^{\hbar },{\mathbb{R}}^{\hbar },S^{A},I^{A},{\left({\mathbb{R}}^{A}\right)}_{1}\right)-{ℷ}_{6}\left(\tau ,S^{\hbar },I^{\hbar },{\mathbb{R}}^{\hbar },S^{A},I^{A},{\left({\mathbb{R}}^{A}\right)}_{2}\right)\right|}^{2}, & \\ & \leq {\left(\Lambda +\omega \right)}^{2}{∥{\left({\mathbb{R}}^{A}\right)}_{1}-{\left({\mathbb{R}}^{A}\right)}_{2}∥}_{\infty }^{2},\\ & \leq {\bar{𝕜}}_{6}{∥{\left({\mathbb{R}}^{A}\right)}_{1}-{\left({\mathbb{R}}^{A}\right)}_{2}∥}_{\infty }^{2}, \end{array} \end{array}$$

where


$$\begin{array}{c} {\bar{𝕜}}_{6}={\left(\Lambda +\omega \right)}^{2}. \end{array}$$

Thus, our model (10) has a unique solution if


$$\begin{array}{c} \max\{\begin{array}{c} \begin{array}{ccc} & \left\{\frac{|\lambda {|}^{2}+|\rho {|}^{2}|I^{A}{|}^{2}}{\left(|\alpha {|}^{2}+|\delta {\mathbb{R}}^{\hbar }{|}^{2}\right)}\right\}, & \\ & \left\{\frac{|\lambda +\zeta {|}^{2}}{|\rho I^{A}S^{\hbar }{|}^{2}}\right\},\\ & \left\{\frac{|\lambda +\delta {|}^{2}}{|\zeta I^{\hbar }{|}^{2}}\right\},\\ & \left\{\frac{|(\Lambda +\eta )I^{A}{|}^{2}}{|\phi {|}^{2}+|\beta {\mathbb{R}}^{A}{|}^{2}}\right\},\\ & \left\{\frac{{\left|\nu^{𝜃}\right|}^{2}}{{\left|\left(\eta^{𝜃}I^{\hbar }+{𝕜}^{𝜃}L\right)S^{A}\right|}^{2}}\right\},\\ & \left\{\frac{|\Lambda +\beta {|}^{2}}{|\omega I^{A}{|}^{2}}\right\} \end{array}<1.\; \end{array} \end{array}$$

### 4.2 Existence with unique solution for FFP

Now, we prove existence and uniqueness of solution for fractal fractional system with power law kernel. For this, first consider the Leptospirosis model (7) in the fractal fractional sense with power law kernel


$$\begin{array}{cccc} {,}^{FFP}D^{\xi ,\gamma }S^{\hbar }(\tau ) & =\alpha -(\lambda +\rho I^{A})S^{\hbar }+\delta {\mathbb{R}}^{\hbar }, & & \\ {,}^{FFP}D^{\xi ,\gamma }I^{\hbar }(\tau ) & =\rho I^{A}S^{\hbar }-(\lambda +\zeta )I^{\hbar }, & & \\ {,}^{FFP}D^{\xi ,\gamma }{\mathbb{R}}^{\hbar }(\tau ) & =\zeta I^{\hbar }-(\lambda +\delta ){\mathbb{R}}^{\hbar }, & \\ {,}^{FFP}D^{\xi ,\gamma }S^{A}(\tau ) & =\phi -(\Lambda +\eta I^{A})S^{A}+\beta {\mathbb{R}}^{A}, & & \\ {,}^{FFP}D^{\xi ,\gamma }I^{A}(\tau ) & =\eta I^{A}S^{A}-(\Lambda +\omega )I^{A}, & & \\ {,}^{FFP}D^{\xi ,\gamma }{\mathbb{R}}^{A}(\tau ) & =\omega I^{A}-(\Lambda +\beta ){\mathbb{R}}^{A}. & & \end{array}$$ (12)

As the integral can be differentiated, so we can express our suggested model in the form:


$$\begin{array}{cccc} {,}^{RL}D^{\xi }S^{\hbar }(\tau ) & =\gamma \tau^{\gamma -1}F_{1}(\tau ,ℶ), & & \\ {,}^{RL}D^{\xi }I^{\hbar }(\tau ) & =\gamma \tau^{\gamma -1}F_{2}(\tau ,ℶ), & & \\ {,}^{RL}D^{\xi }{\mathbb{R}}^{\hbar }(\tau ) & =\gamma \tau^{\gamma -1}F_{3}(\tau ,ℶ), & \\ {,}^{RL}D^{\xi }S^{A}(\tau ) & =\gamma \tau^{\gamma -1}F_{4}(\tau ,ℶ), & & \\ {,}^{RL}D^{\xi }I^{A}(\tau ) & =\gamma \tau^{\gamma -1}F_{5}(\tau ,ℶ), & & \\ {,}^{RL}D^{\xi }{\mathbb{R}}^{A}(\tau ) & =\gamma \tau^{\gamma -1}F_{6}(\tau ,ℶ), & & \end{array}$$ (13)

where


$$\begin{array}{cccc} F_{1} & =\alpha -(\lambda +\rho I^{A})S^{\hbar }+\delta {\mathbb{R}}^{\hbar }, & & \\ F_{2} & =\rho I^{A}S^{\hbar }-(\lambda +\zeta )I^{\hbar }, & & \\ F_{3} & =\zeta I^{\hbar }-(\lambda +\delta ){\mathbb{R}}^{\hbar }, & \\ F_{4} & =\phi -(\Lambda +\eta I^{A})S^{A}+\beta {\mathbb{R}}^{A}, & & \\ F_{5} & =\eta I^{A}S^{A}-(\Lambda +\omega )I^{A}, & & \\ F_{6} & =\omega I^{A}-(\Lambda +\beta ){\mathbb{R}}^{A}. & & \end{array}$$ (14)

and  ℶ  is defined in Eq (11). We can write the above system (12) as


$$\begin{array}{c} {,}^{RL}D^{\xi }\Phi (\tau )=\gamma \tau^{\gamma -1}\Psi (,\tau ,\Phi (\tau )),\Phi (0)=\Phi_{0}. \end{array}$$ (15)

The replacement of ${,}^{RL}D^{\xi ,\gamma }{\text{by}}^{C}D^{\xi ,\gamma }$ and utilizing fractional integral in the Caputo sense, we obtain


$$\Phi (\tau )=\Phi (0)+\frac{\gamma }{\Gamma (\xi )}\int_{0}^{\tau }\phi^{\gamma -1}{\left(\tau -\phi \right)}^{\gamma -1}\Psi (\phi ,\Phi (\phi ))d\phi ,$$

where


Ψ (τ,Φ(τ) )= {F1(τ,ℶ),F2(τ,ℶ),F3(τ,ℶ),F4(τ,ℶ),F5(τ,ℶ),F6(τ,ℶ).^

To prove our existence results, we define a complete norm space = {C×C×C×C×C×C} with norm


∥Φ∥= max ⁡ τ∈[0,⊤ ⁡] |Sℏ(τ)+Iℏ(τ)+ℝℏ(τ)+SA(τ)+IA(τ)+ℝA(τ)|.

We define an operator *Ξ* : →  as


$$\begin{array}{c} \Xi (\Phi (\tau ))=\phi (0)+\frac{\gamma }{\Gamma (\xi )}\int_{0}^{\tau }\phi^{\gamma -1}{\left(\tau -\phi \right)}^{\gamma -1}\Psi (\phi ,\Phi (\phi ))d\phi . \end{array}$$ (16)

Now, we are assuming that the function *Ψ* ( *τ* , *Φ* ( *τ* ) )  always satisfies the two conditions given below:

- For any *Φ* ∈ , there exists two positive constants $\varrho_{1},\varrho_{2}$ which satisfy the following expression:
  $$\begin{array}{c} |\Psi (\tau ,\Phi (\tau ))|\leq \varrho_{1}|\Phi |+\varrho_{2}. \end{array}$$ (17)
- For every $\Phi ,\bar{\Phi }\in$, we have a constant *Π* > 0 such that
  $$\begin{array}{c} |\Psi (\tau ,\Phi (\tau ))-\Psi (\tau ,\bar{\Phi }(\tau ))|\leq \Pi |\Phi (\tau )-\bar{\Phi }(\tau )|. \end{array}$$ (18)

**Theorem 5:** If the condition (17) holds and the function *Ψ* : [ 0 , *⊤* ⁡ ] × → *ℝ* possess continuity, then the model considered (12) has at least one solution.

**Proof:** The continuity of *Ψ* *implies that* *Ξ* is also continuous. It remains to prove that the aforementioned function *Ξ* is completely continuous. Let *Ξ* *For each* *Φ* ∈ , we have


∥Ξ(Φ)∥= max ⁡ τ∈[0,⊤ ⁡] |ϕ(0)+γΓ(ξ) ∫ 0τϕγ−1(τ−ϕ)γ−1Ψ(ϕ,Φ(ϕ))dϕ|,≤ϕ(0)+ max ⁡ τ∈[0,⊤ ⁡]γΓ(ξ) ∫ 0τϕγ−1(τ−ϕ)γ−1 |Ψ(ϕ,Φ(ϕ))|dϕ,≤Φ(0)+γ⊤ ⁡γ+ξ−1Γ(ξ)(ϱ1|Φ|+ϱ2)B(ξ,γ),≤P, (19)

where *B* ( *ξ* , *γ* )  stands for the beta function. This proves the uniform boundedness of *Ξ*.

In the next step, we will prove that *Ξ* is equi-continuous. Consider $\tau_{1}<\tau_{2}\leq ⊤$ such that


$$\begin{array}{cccc} \left|\Xi (\Phi (\tau_{2}))-\Xi (\Phi (\tau_{1}))\right| & =|\frac{\gamma }{\Gamma (\xi )}\int_{0}^{\tau_{2}}\phi^{\gamma -1}{\left(\tau_{2}-\phi \right)}^{\gamma -1}\Psi (\phi ,\Phi (\phi ))d\phi & & \\ & \;-\frac{\gamma }{\Gamma (\xi )}\int_{0}^{\tau_{1}}\phi^{\gamma -1}{\left(\tau_{1}-\phi \right)}^{\gamma -1}\Psi (\phi ,\Phi (\phi ))d\phi |, & & \\ & \leq \frac{\gamma }{\xi }(\varrho_{1}|\Phi |+\varrho_{2})B(\xi ,\gamma )(\tau_{2}^{\xi +\gamma -1}-\tau_{1}^{\xi +\gamma -1}). & & \end{array}$$

Consequently, $|\Xi (\Phi (\tau_{2}))-\Xi (\Phi (\tau_{1}))|\to 0$, when $\tau_{1}\to \tau_{2}$. This proves that *Ξ* is equi-continuous. Thus using theorem of “Arzela-Ascoli”, *Ξ* is completely continuous. Furthermore, by fixed point theorem of Schauder, the suggested model possesses minimum one solution.

**Theorem 6:** If condition (18) holds true, and if


$$\varpi =(\frac{\gamma {⊤}^{\gamma +\xi -1}}{\Gamma (\xi )}B(\xi ,\gamma ))\Pi <1,$$

then the model (12) possess maximum one solution.

**Proof:** For any $\Phi ,\bar{\Phi }$, we have


|Ξ(Φ)−Ξ(Φ)|= |Φ(0)+γΓ(ξ) ∫ 0τϕγ−1(τ−ϕ)γ−1Ψ(ϕ,Φ(ϕ))dϕ− (Φ(0)+γΓ(ξ) ∫ 0τϕγ−1(τ−ϕ)γ−1Ψ(ϕ,Φ¯(ϕ))dϕ ) |,= max ⁡ τ∈[0,⊤ ⁡] |γΓ(ξ) ∫ 0τϕγ−1(τ−ϕ)γ−1 [Ψ(ϕ,Φ(ϕ))−Ψ(ϕ,Φ¯(ϕ))dϕ ]|,≤ [γ⊤ ⁡γ+ξ−1Γ(ξ)B(ξ,γ) ]Π∥Φ−Φ¯∥,≤ϖ∥Φ−Φ¯∥,<∥Φ−Φ¯∥. (20)

So the function *Ξ* fulfills the condition of contraction. Thus uniqueness of the solution implies from “Banach contraction principle”.

### 4.3 Existence with unique solution for FFE

Now, we prove existence of minimum one solution for fractal fractional system with exponential decay kernel. For this, consider the Leptospirosis model (7) in the fractal fractional sense with exponential decay kernel


$$\begin{array}{cccc} {,}^{FFE}D^{\xi ,\gamma }S^{\hbar }(\tau ) & =\alpha -(\lambda +\rho I^{A})S^{\hbar }+\delta {\mathbb{R}}^{\hbar }, & & \\ {,}^{FFE}D^{\xi ,\gamma }I^{\hbar }(\tau ) & =\rho I^{A}S^{\hbar }-(\lambda +\zeta )I^{\hbar }, & & \\ {,}^{FFE}D^{\xi ,\gamma }{\mathbb{R}}^{\hbar }(\tau ) & =\zeta I^{\hbar }-(\lambda +\delta ){\mathbb{R}}^{\hbar }, & \\ {,}^{FFE}D^{\xi ,\gamma }S^{A}(\tau ) & =\phi -(\Lambda +\eta I^{A})S^{A}+\beta {\mathbb{R}}^{A}, & & \\ {,}^{FFE}D^{\xi ,\gamma }I^{A}(\tau ) & =\eta I^{A}S^{A}-(\Lambda +\omega )I^{A}, & & \\ {,}^{FFE}D^{\xi ,\gamma }{\mathbb{R}}^{A}(\tau ) & =\omega I^{A}-(\Lambda +\beta ){\mathbb{R}}^{A}. & & \end{array}$$ (21)

As the integral can be differentiated, so we can express our suggested model in the form:


$$\begin{array}{cccc} {,}^{CF}D^{\xi }S^{\hbar }(\tau ) & =\gamma \tau^{\gamma -1}F_{1}(\tau ,ℶ), & & \\ {,}^{CF}D^{\xi }I^{\hbar }(\tau ) & =\gamma \tau^{\gamma -1}F_{2}(\tau ,ℶ), & & \\ {,}^{CF}D^{\xi }{\mathbb{R}}^{\hbar }(\tau ) & =\gamma \tau^{\gamma -1}F_{3}(\tau ,ℶ), & \\ {,}^{CF}D^{\xi }S^{A}(\tau ) & =\gamma \tau^{\gamma -1}F_{4}(\tau ,ℶ), & & \\ {,}^{CF}D^{\xi }I^{A}(\tau ) & =\gamma \tau^{\gamma -1}F_{5}(\tau ,ℶ), & & \\ {,}^{CF}D^{\xi }{\mathbb{R}}^{A}(\tau ) & =\gamma \tau^{\gamma -1}F_{6}(\tau ,ℶ), & & \end{array}$$ (22)

where $F_{1},F_{2},F_{3},F_{4},F_{5},F_{6}\text{and}ℶ$ are the same as stated in system (11) and (14).

We can write the above system (21) as


$$\begin{array}{c} {,}^{CF}D^{\xi }\Phi (\tau )=\gamma \tau^{\gamma -1}\Psi (\tau ,\Phi (\tau )),\Phi (0)=\Phi_{0}. \end{array}$$ (23)

Using CF integral on the above expression, we obtain


$$\begin{array}{c} \Phi (\tau )=\phi (0)+\frac{\gamma \tau^{\gamma -1}(1-\xi )}{M(\xi )}\Psi (\tau ,\Phi (\tau ))+\frac{\xi \gamma }{M(\xi )}\int_{0}^{\tau }\phi^{\gamma -1}\Psi (\phi ,\Phi (\phi ))d\phi . \end{array}$$ (24)

**Theorem 7:** If the condition (17) holds, then the model considered in (21) has minimum one solution if


$$G=[\frac{\gamma \tau^{\gamma -1}(1-\xi )\Pi }{M(\xi )}+\frac{\xi {⊤}^{\gamma }\Pi }{M(\xi )}]<1.$$

**Proof:** To prove our required result, we define the Picard operator *K* : →  as


$$\begin{array}{c} K(\Phi (\tau ))=\Phi (0)+\frac{\gamma \tau^{\gamma -1}(1-\xi )}{M(\xi )}\Psi (\tau ,\Phi (\tau ))+\frac{\xi \gamma }{M(\xi )}\int_{0}^{\tau }\phi^{\gamma -1}\Psi (\phi ,\Phi (\phi ))d\phi . \end{array}$$ (25)

In the First step, we aim to prove that the solution of the system (21) is bounded


$$\begin{array}{llll} ∥K(\Phi (\tau ))-\Phi (0)∥ & =∥\Phi (0)+\frac{\gamma \tau^{\gamma -1}(1-\xi )}{M(\xi )}\Psi (\tau ,\Phi (\tau ))\; & & \;\\ & \;+\frac{\xi \gamma }{M(\xi )}\int_{0}^{\tau }\phi^{\gamma -1}\Psi (\phi ,\Phi (\phi ))d\phi -\Phi (0)∥,\; & & \;\\ & \leq ∥\frac{\gamma \tau^{\gamma -1}(1-\xi )}{M(\xi )}\Psi (\tau ,\Phi (\tau ))+\frac{\xi \gamma }{M(\xi )}\int_{0}^{\tau }\phi^{\gamma -1}\Psi (\phi ,\Phi (\phi ))d\phi ∥,\; & & \;\\ & \leq ∥\frac{\gamma \tau^{\gamma -1}(1-\xi )}{M(\xi )}\Psi (\tau ,\Phi (\tau ))∥+∥\frac{\xi \gamma }{M(\xi )}\int_{0}^{\tau }\phi^{\gamma -1}\Psi (\phi ,\Phi (\phi ))d\phi ∥,\; & & \;\\ & \leq \frac{\gamma \tau^{\gamma -1}(1-\xi )}{M(\xi )}∥\Psi (\tau ,\Phi (\tau ))∥+\frac{\xi \gamma }{M(\xi )}\int_{0}^{\tau }\phi^{\gamma -1}∥\Psi (\phi ,\Phi (\phi ))∥d\phi ,\; & & \;\\ & \leq \frac{\gamma \tau^{\gamma -1}(1-\xi )}{M(\xi )}(\varrho_{1}|\Phi |+\varrho_{2})+\frac{\xi {⊤}^{\gamma }\Pi }{M(\xi )}(\varrho_{1}|\Phi |+\varrho_{2}),\; & & \;\\ & \leq [\frac{\gamma \tau^{\gamma -1}(1-\xi )\Pi }{M(\xi )}+\frac{\xi {⊤}^{\gamma }\Pi }{M(\xi )}](\varrho_{1}|\Phi |+\varrho_{2}),\; & & \;\\ & <\infty .\; & & \; \end{array}$$

Now, we check the contraction condition.


$$\begin{array}{cccc} ∥K(\Phi_{1}(\tau ))-K(\Phi_{2}(\tau ))∥ & =∥\frac{\gamma \tau^{\gamma -1}(1-\xi )}{M(\xi )}\Psi (\tau ,\Phi_{1}(\tau ))+\frac{\xi \gamma }{M(\xi )}\int_{0}^{\tau }\phi^{\gamma -1}\Psi (\phi ,\Phi_{1}(\phi ))d\phi & & \\ & \;-\frac{\gamma \tau^{\gamma -1}(1-\xi )}{M(\xi )}\Psi (\tau ,\Phi_{2}(\tau ))-\frac{\xi \gamma }{M(\xi )}\int_{0}^{\tau }\phi^{\gamma -1}\Psi (\phi ,\Phi_{2}(\phi ))d\phi ∥, & & \\ & =∥\frac{\gamma \tau^{\gamma -1}(1-\xi )}{M(\xi )}(\Psi (\tau ,\Phi_{1}(\tau ))-\Psi (\tau ,\Phi_{2}(\tau )))d\phi & & \\ & \;+\frac{\xi \gamma }{M(\xi )}\int_{0}^{\tau }\phi^{\gamma -1}(\Psi (\phi ,\Phi_{2}(\phi ))-\Psi (\phi ,\Phi_{2}(\phi )))d\phi ∥, & \\ & \leq \frac{\gamma \tau^{\gamma -1}(1-\xi )\Pi }{M(\xi )}∥\Phi_{1}(\tau ))-\Phi_{2}(\tau ))∥+\frac{\xi {⊤}^{\gamma }\Pi }{M(\xi )}∥\Phi_{1}(\tau ))-\Phi_{2}(\tau ))∥, & & \\ & \leq [\frac{\gamma \tau^{\gamma -1}(1-\xi )\Pi }{M(\xi )}+\frac{\xi {⊤}^{\gamma }\Pi }{M(\xi )}]∥\Phi_{1}(\tau )-\Phi_{2}(\tau )∥, & & \\ & \leq G∥\Phi_{1}(\tau )-\Phi_{2}(\tau )∥. & & \end{array}$$ (26)

Since both conditions are fulfilled, so by “Banach contraction theorem”, the model (21) possess maximum one solution.

## 5 Hyers Ulam stability

Hyers Ulam stability, also knows as Hyers Ulam Rassias stability, is an important type of stability used in various branches of Science introduced by [53,54]. Three mathematicians : David Hyers, Stanislaw Ulam, and Themistocles Rassias are credited to establish this concept to find the stability of the Cauchy equation. It states that a function is a solution of a functional equation if it approaches to the solution. It includes a vast variety of applications including nonlinear functional Eqs [55,56].

### 5.1 Hyers Ulam stability for FFP

In this subsection, we will discuss the Hyers Ulam stability of the model for fractal fractional derivative using power law kernel. Same can be deduced for fractal fractional exponential decay case sense by following the similar pattern.

**Definition 7:** The suggested model possess Hyers Ulam stability if we have non negative $\Xi_{\xi ,\gamma }$ s. that for all *K* : → , and for all *Φ* ∈ *ℂ* ( [ 0 , *⊤* ⁡ ] , *ℝ* )  which satisfies the inequality given by:


$${|}^{FFP}D^{\xi ,\gamma }\Phi (\tau )-\Psi (\tau ,\Phi (\tau ))|\leq 𝜖,\tau \in [0,⊤],$$

and *∃* ⁡  a unique solution *U* ∈ *ℂ* ( [ 0 , *⊤* ⁡ ] , *ℝ* )  s.that


$$|\Phi (\tau )-U(\tau )|\leq \Xi_{\xi ,\omega }𝜖,\tau \in [0,⊤].$$

Let *Φ* ∈ *ℂ* [ 0 , *⊤* ⁡ ]  be a small perturbation with *Φ* ( 0 ) = 0 .  Let

- *Φ* ( 0 ) = 0 . ,
-
${,}^{FFP}D^{\xi ,\gamma }\Phi (\tau )=\Psi (\tau ,\Phi (\tau ))+\Phi (\tau ).$

**Lemma 2:** The following perturbed solution


$${,}^{FFP}D^{\xi ,\gamma }\Phi (\tau )=\Psi (\tau ,\Phi (\tau ))+\Phi (\tau ),\Phi (0)=\Phi_{0}.$$

possess the solution which satisfies the following expression


$$\left|\phi (\tau )-(\Phi (0)+\frac{\gamma }{\Gamma (\xi )}\int_{0}^{\tau }\phi^{\gamma -1}{\left(\tau -\phi \right)}^{\gamma -1}\Psi (\tau ,\Phi (\phi ))d\phi )\right|\leq {\bar{𝜗}}_{\xi ,\gamma }𝜖,$$

where ${\bar{𝜗}}_{\xi ,\gamma }=\frac{\gamma }{\Gamma (\xi )}{⊤}^{\xi +\gamma -1}B(\xi ,\gamma ).$

**Proof:** The proof is simple and left for reader.

**Lemma 3:** Let both condition (18) is satisfied and if *ϖ* < 1 ,  then our suggested model (12) possess Hyers Ulam stability.

**Proof:** As we have already proved the uniqueness of the solution of our model, so we assume *U* ∈  as any other solution, this implies


$$\begin{array}{cccc} \left|\Phi (\tau )-U(\tau )\right| & =\left|\Phi (\tau )-(U(0)+\frac{\gamma }{\Gamma (\xi )}\int_{0}^{\tau }\phi^{\gamma -1}{\left(\tau -\phi \right)}^{\gamma -1}\Psi (\tau ,U(\phi ))d\phi )\right|, & & \\ & \leq \left|\Phi (\tau )-(\Phi (0)+\frac{\gamma }{\Gamma (\xi )}\int_{0}^{\tau }\phi^{\gamma -1}{\left(\tau -\phi \right)}^{\gamma -1}\Psi (\tau ,\Phi (\phi ))d\phi )\right| & & \\ & \;+\left|\Phi (0)+\frac{\gamma }{\Gamma (\xi )}\int_{0}^{\tau }\phi^{\gamma -1}{\left(\tau -\phi \right)}^{\gamma -1}\Psi (\tau ,\Phi (\phi ))d\phi \right| & & \\ & \;-\left|U(0)+\frac{\gamma }{\Gamma (\xi )}\int_{0}^{\tau }\phi^{\gamma -1}{\left(\tau -\phi \right)}^{\gamma -1}\Psi (\tau ,U(\phi ))d\phi \right|, & \\ & \leq {\bar{𝜗}}_{\xi ,\gamma }𝜖+(\frac{\gamma }{\Gamma (\xi )}{⊤}^{\xi +\gamma -1}B(\xi ,\gamma ))\Pi \left|\Phi (\tau )-U(\tau )\right|, & & \\ & \leq {\bar{𝜗}}_{\xi ,\gamma }𝜖+\varpi |\Phi (\tau )-U(\tau )|. & & \end{array}$$ (27)

Thus we obtain


$$∥\Phi (\tau )-U(\tau )∥\leq {\bar{𝜗}}_{\xi ,\gamma }𝜖+\varpi ∥\Phi (\tau )-U(\tau )∥.$$

From the above expression, we have


$$∥\Phi (\tau )-U(\tau )∥\leq \Xi_{\xi ,\omega }𝜖,$$

where $\Xi_{\xi ,\omega }=\frac{{\bar{𝜗}}_{\xi ,\gamma }}{1-\varpi }.$

### 5.2 Hyers Ulam stability for FFM

**Definition 8:** The Leptospirosis model in the fractal fractional sense (10) is called Hyers Ulam stable if $\exists \;\text{a}\;\text{constant}\;\Theta_{i}>0,i=1,2,\cdots 6\;\text{which}\;\text{satisfy}\;\text{for}\;\text{every}\;\varkappa_{i}>0,i=1,2,\cdots 6.$


$$\begin{array}{llll} & ∥S^{\hbar }(\tau )-\frac{\gamma \xi }{AB(\xi )\Gamma (\xi )}\int_{0}^{\tau }{\left(\tau -\phi \right)}^{\xi -1}\phi^{\gamma -1}F_{1}\left(\phi ,S^{\hbar }\right)d\phi -\frac{\gamma (1-\xi )\tau^{\gamma -1}}{AB(\xi )}F_{1}\left(\tau ,S^{\hbar }\right)∥\leq \varkappa_{1},\; & & \;\\ , & ∥I^{\hbar }(\tau )-\frac{\gamma \xi }{AB(\xi )\Gamma (\xi )}\int_{0}^{\tau }{\left(\tau -\phi \right)}^{\xi -1}\phi^{\gamma -1}F_{2}\left(\phi ,I^{\hbar }\right)d\phi -\frac{\gamma (1-\xi )\tau^{\gamma -1}}{AB(\xi )}F_{2}\left(\tau ,I^{\hbar }\right)∥\leq \varkappa_{2},\; & & \;\\ & ∥{\mathbb{R}}^{\hbar }(\tau )-\frac{\gamma \xi }{AB(\xi )\Gamma (\xi )}\int_{0}^{\tau }{\left(\tau -\phi \right)}^{\xi -1}\phi^{\gamma -1}F_{3}\left(\phi ,{\mathbb{R}}^{\hbar }\right)d\phi -\frac{\gamma (1-\xi )\tau^{\gamma -1}}{AB(\xi )}F_{3}\left(\tau ,{\mathbb{R}}^{\hbar }\right)∥\leq \varkappa_{3},\; & & \;\\ & ∥S^{A}(\tau )-\frac{\gamma \xi }{AB(\xi )\Gamma (\xi )}\int_{0}^{\tau }{\left(\tau -\phi \right)}^{\xi -1}\phi^{\gamma -1}F_{4}\left(\phi ,S^{A}\right)d\phi -\frac{\gamma (1-\xi )\tau^{\gamma -1}}{AB(\xi )}F_{4}\left(\tau ,S^{A}\right)∥\leq \varkappa_{4},\; & & \;\\ & ∥I^{A}(\tau )-\frac{\gamma \xi }{AB(\xi )\Gamma (\xi )}\int_{0}^{\tau }{\left(\tau -\phi \right)}^{\xi -1}\phi^{\gamma -1}F_{5}\left(\phi ,I^{A}\right)d\phi -\frac{\gamma (1-\xi )\tau^{\gamma -1}}{AB(\xi )}F_{5}\left(\tau ,I^{A}\right)∥\leq \varkappa_{5},\; & & \;\\ & ∥{\mathbb{R}}^{A}(\tau )-\frac{\gamma \xi }{AB(\xi )\Gamma (\xi )}\int_{0}^{\tau }{\left(\tau -\phi \right)}^{\xi -1}\phi^{\gamma -1}F_{6}\left(\phi ,{\mathbb{R}}^{A}\right)d\phi -\frac{\gamma (1-\xi )\tau^{\gamma -1}}{AB(\xi )}F_{6}\left(\tau ,{\mathbb{R}}^{A}\right)∥\leq \varkappa_{6}.\; & & \; \end{array}$$

*∃* ⁡  approximate solutions of the Leptospirosis model (10) $\bar{S^{\hbar }},\bar{I^{\hbar }},\bar{{\mathbb{R}}^{\hbar }},\bar{S^{A}},\bar{I^{A}},\bar{{\mathbb{R}}^{A}}$ which satisfy the given model:


$$\begin{array}{cccc} ∥S^{\hbar }(\tau )-\bar{S^{\hbar }}(\tau )∥ & =\frac{\gamma \xi }{AB(\xi )\Gamma (\xi )}\int_{0}^{\tau }{\left(\tau -\phi \right)}^{\xi -1}\phi^{\gamma -1}∥F_{1}\left(\phi ,S^{\hbar }\right)-F_{1}\left(\phi ,\bar{S^{\hbar }}\right)∥d\phi & & \\ & +\frac{\gamma (1-\xi )\tau^{\gamma -1}}{AB(\xi )}∥F_{1}\left(\tau ,S^{\hbar }\right)-F_{1}\left(\tau ,\bar{S^{\hbar }}\right)∥, & & \\ & \leq [\frac{\xi \gamma \Gamma (\gamma )}{AB(\xi )\Gamma (\xi +\gamma )}+\frac{\gamma (1-\xi )}{AB(\xi )}]\Pi_{1}∥S^{\hbar }-\bar{S^{\hbar }}∥. & & \end{array}$$

We let $\Upsilon_{1}=[\frac{\xi \gamma \Gamma (\gamma )}{AB(\xi )\Gamma (\xi +\gamma )}+\frac{\gamma (1-\xi )}{AB(\xi )}]∥S^{\hbar }-\bar{S^{\hbar }}∥.$ Also letting $\Psi_{1}=\Pi_{1}$ transforms the above inequality in the form $∥S^{\hbar }(\tau )-\bar{S^{\hbar }}(\tau )∥\leq \Psi_{1}\Upsilon_{1}.$ Similarly, for other variables, we have


$$\begin{array}{cccc} ∥I^{\hbar }(\tau )-\bar{I^{\hbar }}(\tau )∥ & \leq \Psi_{2}\Upsilon_{2}, & & \\ ∥{\mathbb{R}}^{\hbar }(\tau )-\bar{{\mathbb{R}}^{\hbar }}(\tau )∥ & \leq \Psi_{3}\Upsilon_{3}, & & \\ ∥S^{A}(\tau )-\bar{S^{A}}(\tau )∥ & \leq \Psi_{4}\Upsilon_{4}, & \\ ∥I^{A}(\tau )-\bar{I^{A}}(\tau )∥ & \leq \Psi_{5}\Upsilon_{5}, & & \\ |{\mathbb{R}}^{A}(\tau )-\bar{{\mathbb{R}}^{A}}(\tau )∥ & \leq \Psi_{6}\Upsilon_{6}. & & \end{array}$$ (28)

**Theorem 8:** If the assumptions given above hold true, then the Leptospirosis model (10) is Hyers Ulam stable.

**Proof:** We have already proved that our suggested model (10) has unique solution. Let the model has $\bar{S^{\hbar }},\bar{I^{\hbar }},\bar{{\mathbb{R}}^{\hbar }},\bar{S^{A}},\bar{I^{A}},\bar{{\mathbb{R}}^{A}}$ as the approximate solutions which satisfies the model (10), then we have


$$\begin{array}{cccc} ∥S^{\hbar }(\tau )-\bar{S^{\hbar }}(\tau )∥ & =\frac{\gamma \xi }{AB(\xi )\Gamma (\xi )}\int_{0}^{\tau }{\left(\tau -\phi \right)}^{\xi -1}\phi^{\gamma -1}∥F_{1}\left(\phi ,S^{\hbar }\right)-F_{1}\left(\phi ,\bar{S^{\hbar }}\right)∥d\phi & & \\ & \;+\frac{\gamma (1-\xi )\tau^{\gamma -1}}{AB(\xi )}∥F_{1}\left(\tau ,S^{\hbar }\right)-F_{1}\left(\tau ,\bar{S^{\hbar }}\right)∥, & & \\ & \leq [\frac{\xi \gamma \Gamma (\gamma )}{AB(\xi )\Gamma (\xi +\gamma )}+\frac{\gamma (1-\xi )}{AB(\xi )}]\Pi_{1}∥S^{\hbar }-\bar{S^{\hbar }}∥. & & \end{array}$$

Let ${ℷ}_{1}=[\frac{\xi \gamma \Gamma (\gamma )}{AB(\xi )\Gamma (\xi +\gamma )}+\frac{\gamma (1-\xi )}{AB(\xi )}]∥S^{\hbar }-\bar{S^{\hbar }}∥$ , and $\Pi_{1}=\varkappa_{1}$. So that the inequality stated above takes the shape


$$∥S^{\hbar }(\tau )-\bar{S^{\hbar }}(\tau )∥\leq {ℷ}_{1}\varkappa_{1}.$$

Similarly, for other variables, we have


$$\begin{array}{cccc} ∥I^{\hbar }(\tau )-\bar{I^{\hbar }}(\tau )∥ & \leq {ℷ}_{2}\varkappa_{2}, & & \\ ∥{\mathbb{R}}^{\hbar }(\tau )-\bar{{\mathbb{R}}^{\hbar }}(\tau )∥ & \leq {ℷ}_{3}\varkappa_{3}, & & \\ ∥S^{A}(\tau )-\bar{S^{A}}(\tau )∥ & \leq {ℷ}_{4}\varkappa_{4}, & \\ ∥I^{A}(\tau )-\bar{I^{A}}(\tau )∥ & \leq {ℷ}_{5}\varkappa_{5}, & & \\ ∥{\mathbb{R}}^{A}(\tau )-\bar{{\mathbb{R}}^{A}}(\tau )∥ & \leq {ℷ}_{6}\varkappa_{6}. & & \end{array}$$ (29)

So by definition, the Leptospirosis model (10) possess Hyers Ulam stability, which completes the proof.

## 6 Flip bifurcation

Bifurcation is an important tool for studying dynamic systems, which reveals how variation in parameters change the variables of the system. It helps to identify critical values, stability property and existence of multiple solutions. [57] was the mathematician who first used the term bifurcation. This part analyzes the stability of equilibrium points through bifurcation, that is, what will be the impact of specific parameters change on the stability. For this, we are using the approach from [58].

From our model (7), note that none of the eigen values is 1 − 1, which implies the existence of flip bifurcation if the collection of constants  ( *α* , *λ* , *ρ* , *δ* , *ζ* , *ϕ* , *Λ* , *η* , *β* , *ω* )  lie in the set


$$\begin{array}{c} F{|}_{E^{0}(\frac{\alpha }{\lambda },0,0,\frac{\phi }{\Lambda },0,0)}=\left\{(\alpha ,\lambda ,\rho ,\delta ,\zeta ,\phi ,\Lambda ,\eta ,\beta ,\omega ):\lambda =-\delta ,\Lambda =-\beta \right\}. \end{array}$$ (30)

But we will prove that our model does not possess flip bifurcation at $E^{0}(\frac{\alpha }{\lambda },0,0,\frac{\phi }{\Lambda },0,0)$ if the collection of constants

$(\alpha ,\lambda ,\rho ,\delta ,\zeta ,\phi ,\Lambda ,\eta ,\beta ,\omega )\;\text{lie}\;\text{in}\;F{|}_{E^{0}(\frac{\alpha }{\lambda },0,0,\frac{\phi }{\Lambda },0,0)}.$

**Theorem 9:** The Leptospirosis model (7) does not possess flip bifurcation at $E^{0}(\frac{\alpha }{\lambda },0,0,\frac{\phi }{\Lambda },0,0)$ if the collection of constants

$(\alpha ,\lambda ,\rho ,\delta ,\zeta ,\phi ,\Lambda ,\eta ,\beta ,\omega )\text{lie}\text{in}F{|}_{E^{0}(\frac{\alpha }{\lambda },0,0,\frac{\phi }{\Lambda },0,0)}.$

**Proof:** Since our proposed model (7) remains unchanged with respect to $I_{0}^{\hbar }=R_{0}^{\hbar }=I_{0}^{A}=R_{0}^{A}=0$. That’s why, to ensure the existence of flip bifurcation, we put $I_{0}^{\hbar }=R_{0}^{\hbar }=I_{0}^{A}=R_{0}^{A}=0$ and so we have


$$\begin{array}{c} \begin{array}{cc} S^{\hbar }(\tau ) & =\hbar \alpha +\left(-\hbar \lambda +1\right)S^{\hbar }(\tau ),\\ S^{A}(\tau ) & =\hbar \phi +\left(-\hbar \Lambda +1\right)S^{A}(\tau ). \end{array} \end{array}$$ (31)

Eq (31) can be expressed in the following form:


$$\begin{array}{c} \begin{array}{cc} f(S^{\hbar }(\tau )) & =\hbar \alpha +\left(-\hbar \lambda +1\right)S^{\hbar }(\tau ),\\ g(S^{A}(\tau )) & =\hbar \phi +\left(-\hbar \Lambda +1\right)S^{A}(\tau ). \end{array} \end{array}$$ (32)

Now, taking the partial derivative of the above equations and utilizing the values $\lambda =-\delta ,S_{0}^{\hbar }(\tau )=\frac{\alpha }{\lambda },S_{0}^{A}(\tau )=\frac{\phi }{\Lambda },\Lambda =-\beta$ in Eq (32), we obtain the following


$$\begin{array}{c} \begin{array}{cc} \frac{\partial f(S^{\hbar }(\tau ))}{\partial S^{\hbar }(\tau )}{|}_{\lambda =-\delta ,S_{0}^{\hbar }(\tau )=\frac{\alpha }{\lambda }}= & \hbar \delta +1,\\ \frac{\partial g(S^{A}(\tau ))}{\partial S^{A}(\tau )}{|}_{S_{0}^{A}(\tau )=\frac{\phi }{\Lambda },\Lambda =-\beta }= & 1+\hbar \beta . \end{array} \end{array}$$ (33)

Now, applying partial derivative on $f(S^{\hbar }(\tau ))$ with respect to *λ* and putting the values of $S_{0}^{\hbar }(\tau )\;\text{and}\;\lambda$, we obtain $-\hbar \frac{\alpha }{\lambda }\neq 0$. Similarly, applying the partial derivative on $f(S^{A}(\tau ))$ with respect to *Λ* and putting the values of $S_{0}^{A}(\tau )\;\text{and}\;\Lambda$, we have $-\hbar \frac{\phi }{\Lambda }\neq 0.$ Differentiating Eq (33) again, we obtain


$$\begin{array}{c} \begin{array}{cc} \frac{\partial^{2}f(S^{\hbar }(\tau ))}{\partial S^{2\hbar }(\tau )}= & 0,\\ \frac{\partial^{2}g(S^{A}(\tau ))}{\partial S^{2A}(\tau )}= & 0. \end{array} \end{array}$$ (34)

**Fig 2:**
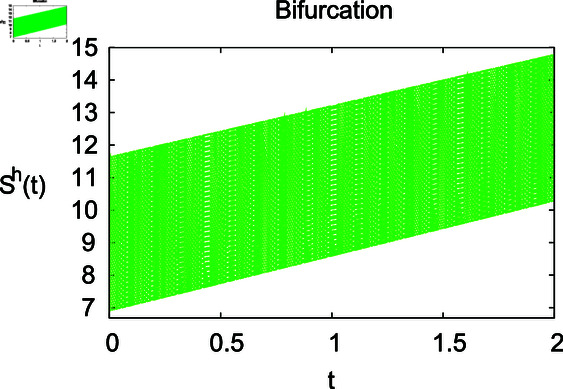
Impact of growth rate *α* on the susceptible population $S^{\hbar }$.

**Fig 3:**
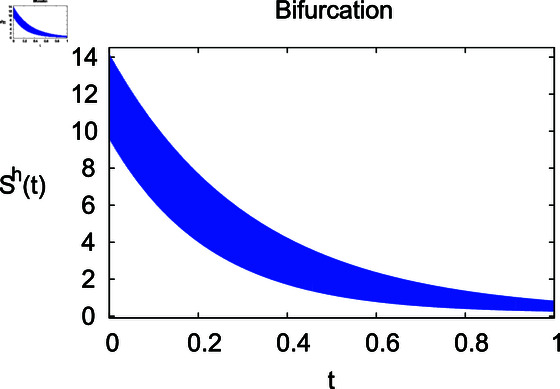
Influence of contact rate *ρ* on vulnerable human population $S^{\hbar }$.

**Fig 4:**
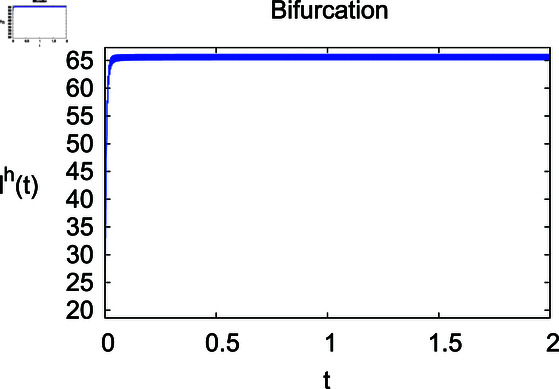
Infected humans $I^{\hbar }$ under infection rate *ρ.*

**Fig 5:**
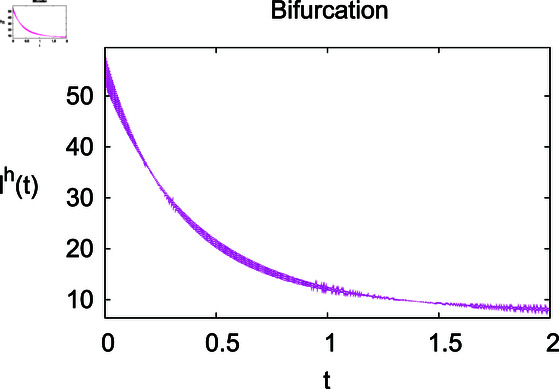
Infected humans $I^{\hbar }$ under recovery rate *ζ.*

**Fig 6:**
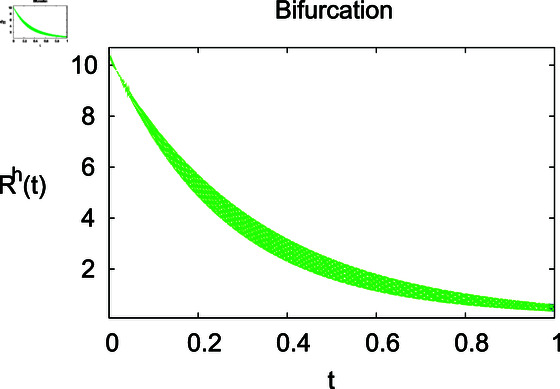
Influence of relapse rate *δ* on recovered humans ${\mathbb{R}}^{\hbar }$.

**Fig 7:**
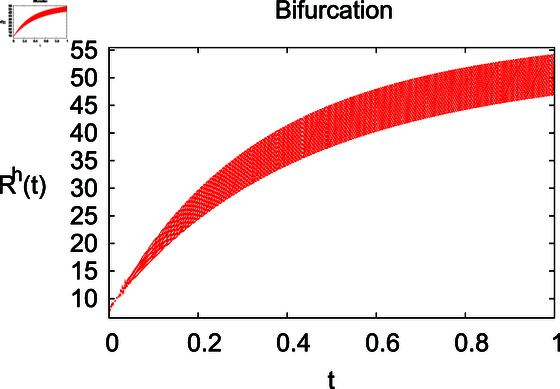
Recovered humans under ${\mathbb{R}}^{\hbar }$ under recovery rate *ζ.*

**Fig 8:**
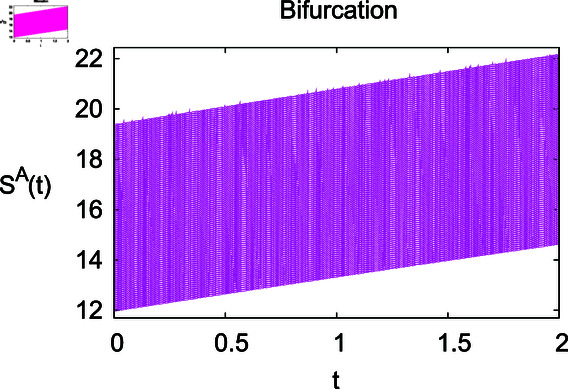
Impact of inflow rate *ϕ* on susceptible animals $S^{A}$

**Fig 9:**
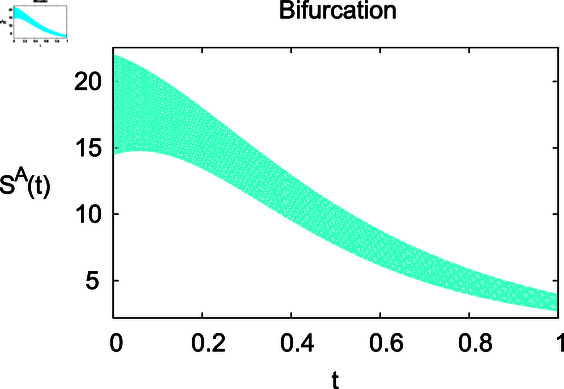
Effect of death rate *Λ* on susceptible animals $S^{A}$ under parameter *Λ.*

**Fig 10:**
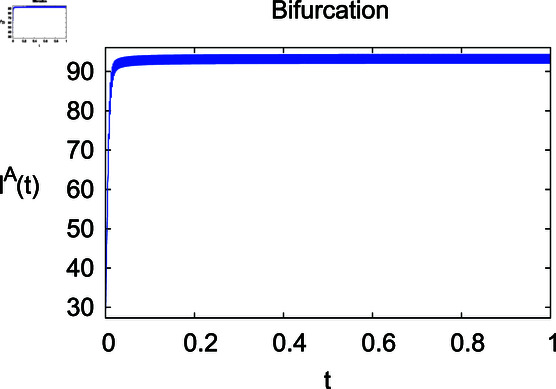
Infected animals $I^{A}$ under *η.*

**Fig 11:**
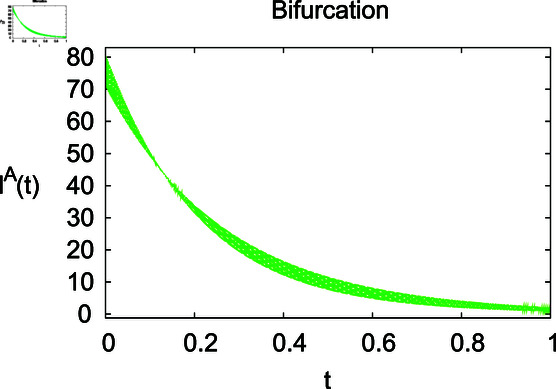
Behaviour of infected animals $I^{A}$ under mortality rate *Λ.*

**Fig 12:**
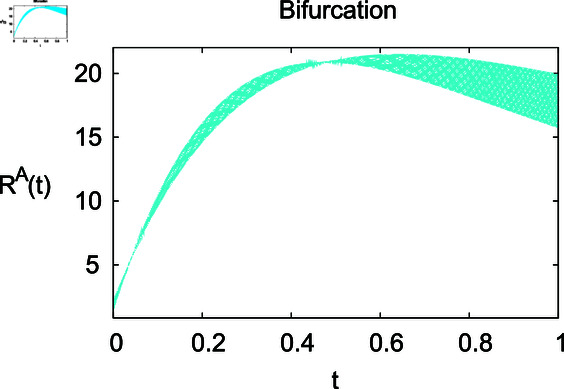
Influence of recovery rate *ω* on recovered animals population ${\mathbb{R}}^{A}$.

**Fig 13:**
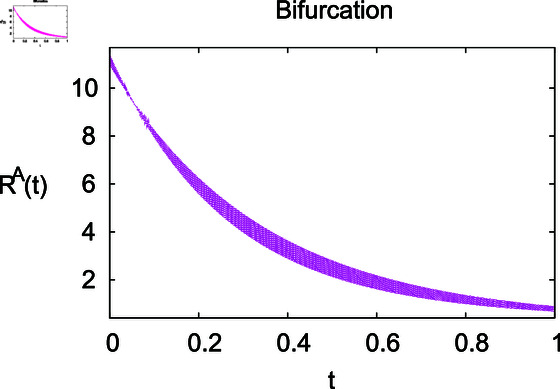
Behaviour of recovered animals ${\mathbb{R}}^{A}$ under relapse rate *β.*

The results mentioned above demonstrate that flip bifurcation is not present for our model (7), since the condition (34) is insufficient to meet the requirement for flip bifurcation existence if the collection of constants  ( *α* , *λ* , *ρ* , *δ* , *ζ* , *ϕ* , *Λ* , *η* , *β* , *ω* )  liein $E^{0}(\frac{\alpha }{\lambda },0,0,\frac{\phi }{\Lambda },0,0).$

**Fig 14:**
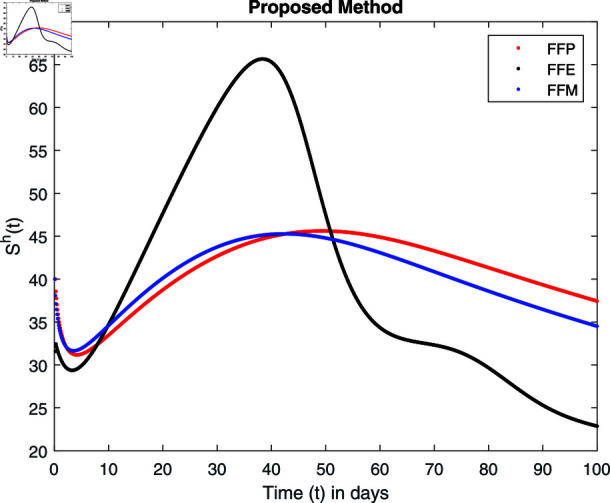
Simulation with fractional value 0.8 and fractal dimension 1.

For these graphs, the parameter values employed are *α* = 1.6, *λ* = 0.000046, *ρ* = 0.0089, *δ* = 0.066, *ζ* = 0.0027, *ϕ* = 1.9, *Λ* = 0.0018, *η* = 0.0079, *β* = 0.05, *ω* = 0.7. Linearization technique is used to achieve the stability and boundedness of Leptospirosis model in Figs 2, 3, 4, 5, 6, 7, 8, 9, 10, 11, 12, and 13. Our theocratical results are supported by Figs 2, 3, 4, 5, 6, 7, 8, 9, 10, 11, 12, and 13, with the help of time steady graphs. In Figs Figs 2, 3, and 4, the effect of parameters *αρ* on susceptible and infected humans have been plotted. The graphs show that by increasing recruitment rate *α*, susceptible humans increase, while increasing contact rate of susceptible humans with infected animals *ρ*, vulnerable humans start to move into infected humans compartment. As a result, susceptible humans start to decrease and infected humans start to increase which is shown in Figs 3 and 4. In Fig 5, one can see that by increasing recovery rate *ζ*, infected humans tend to decrease. In Fig 6, the increment in relapse rate *δ* decrease recovered humans, while increasing recovery rate *ζ* rises recovered humans as shown in Fig 7. Increase of inflow rate of susceptible animals *ϕ* rises susceptible animals population as depicted in Fig 8, while rise of death rate of animals *Λ* declines susceptible animals population as plotted in Fig 9. Fig 10 is devoted to show that increment in infection rate also increases infected animals population, while death rate of infected animals *Λ* has a negative impact on the growth of infected animals compartment as shown in Fig 11. Fig 12 shows that by increasing recovery rate *ω*, population of recovered animals also rises, while relapse rate *β* imposes has a negative influence on the recovered animals as in Fig 13.

**Fig 15:**
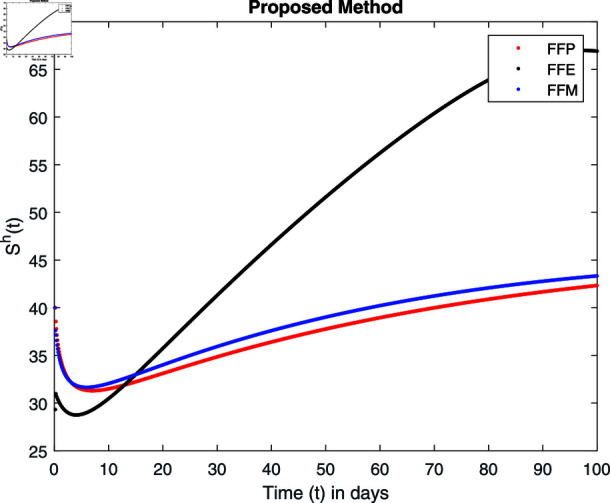
Simulation with fractional value 0.8 and fractal dimension 0.8.

**Fig 16:**
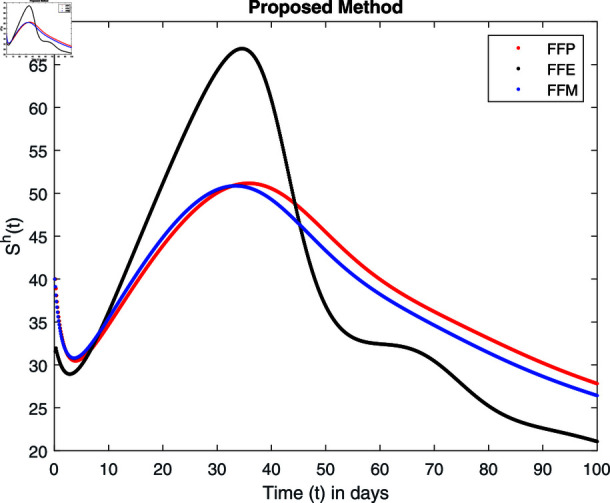
Simulation with fractional value 0.9 and fractal dimension 1.

**Fig 17:**
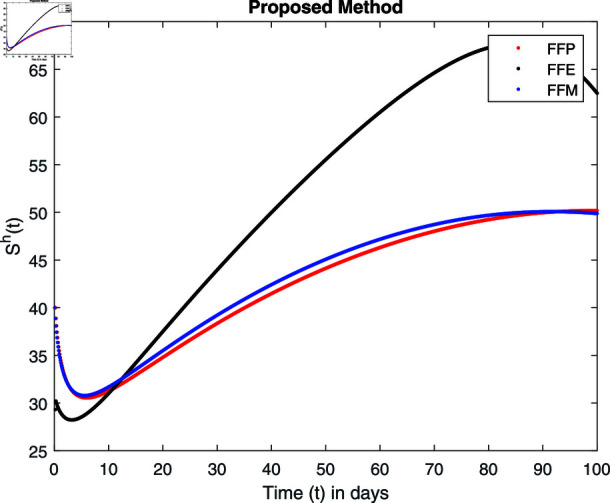
Simulation with fractional value 0.9 and fractal dimension 0.8.

**Fig 18:**
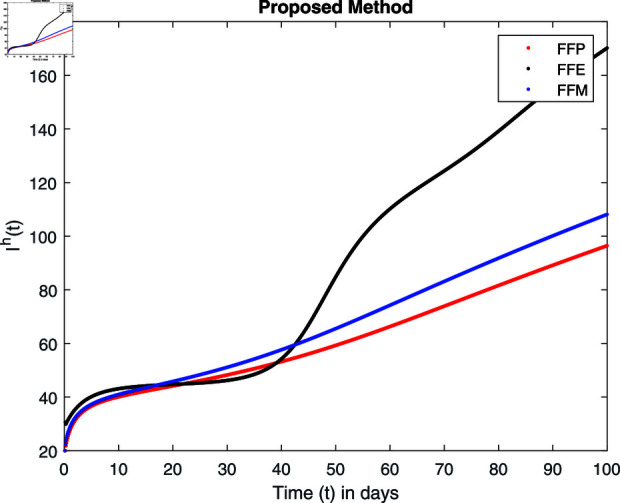
Simulation with fractional value 0.8 and fractal dimension 1.

**Fig 19:**
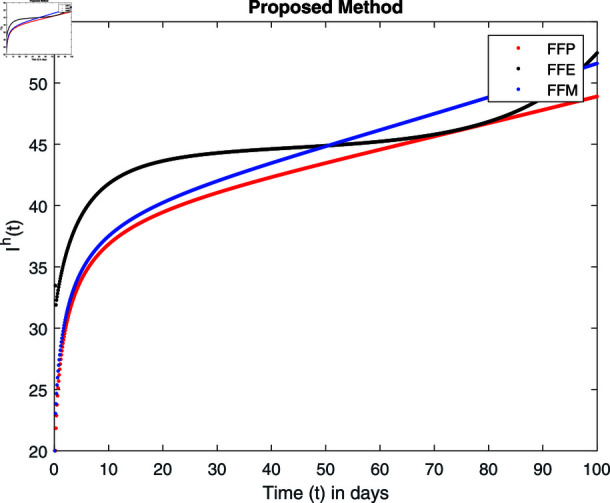
Simulation with fractional value 0.8 and fractal dimension 0.8.

**Fig 20:**
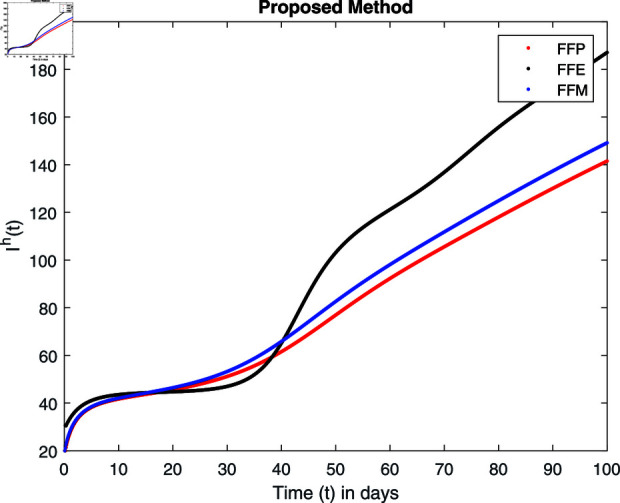
Simulation with fractional value 0.9 and fractal dimension 1.

**Fig 21:**
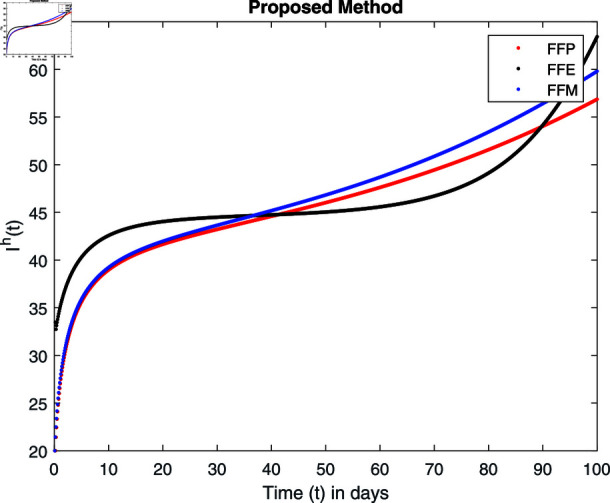
Simulation with fractional value 0.9 and fractal dimension 0.8.

**Fig 22:**
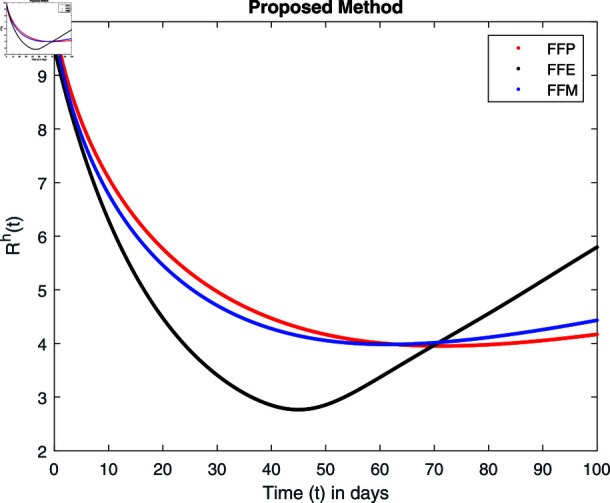
Simulation with fractional value 0.8 and fractal dimension 1.

**Fig 23:**
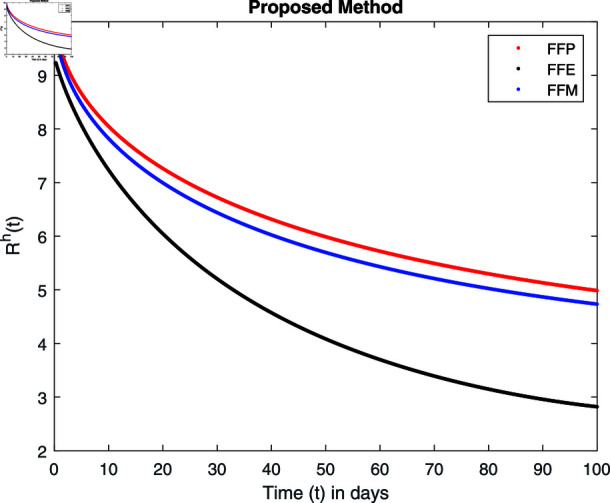
Simulation with fractional value 0.8 and fractal dimension 0.8.

**Fig 24:**
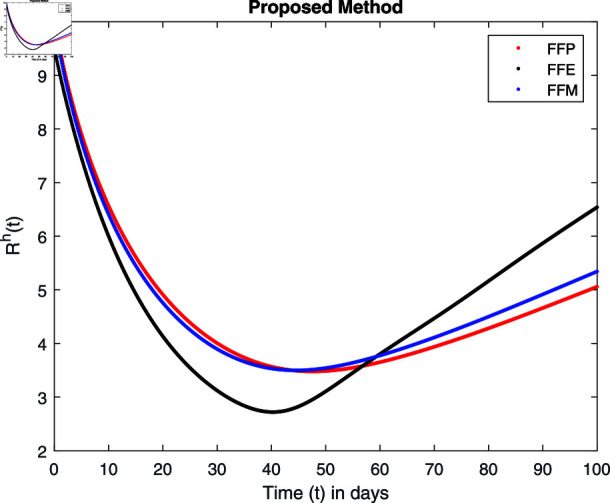
Simulation with fractional value 0.9 and fractal dimension 1.

**Fig 25:**
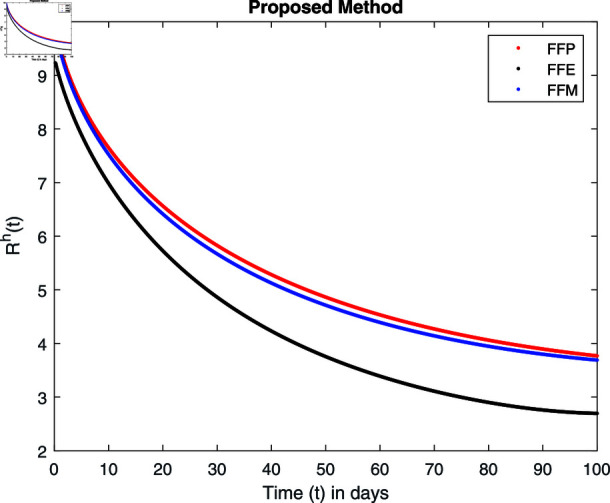
Simulation with fractional value 0.9 and fractal dimension 0.8.

**Fig 26:**
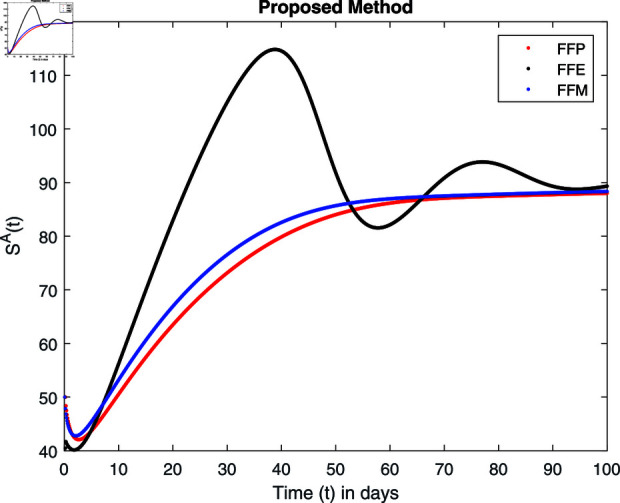
Simulation with fractional value 0.8 and fractal dimension 1.

**Fig 27:**
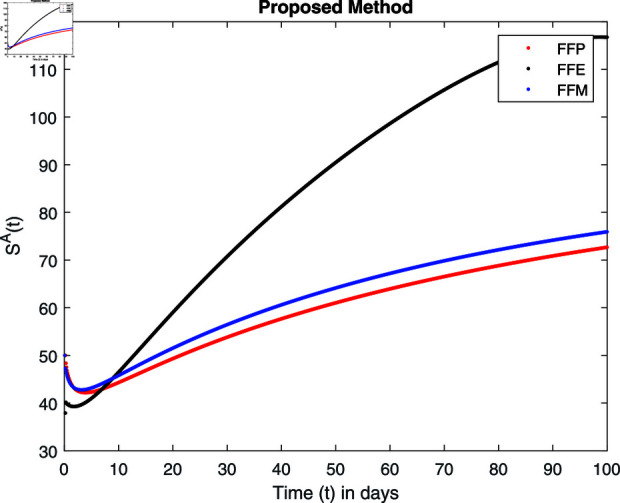
Simulation with fractional value 0.8 and fractal dimension 0.8.

**Fig 28:**
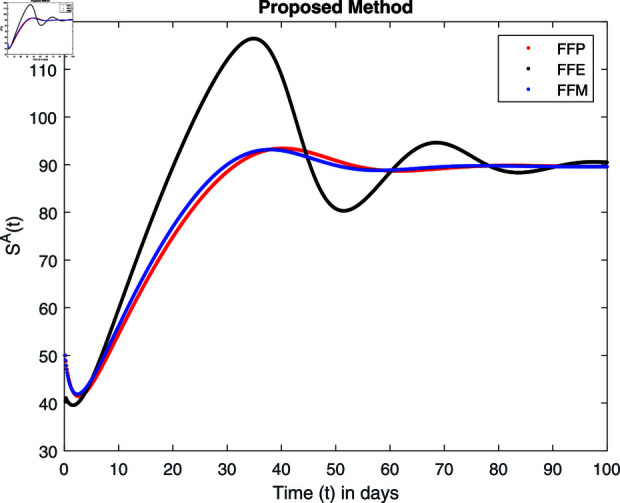
Simulation with fractional value 0.9 and fractal dimension 1.

**Fig 29:**
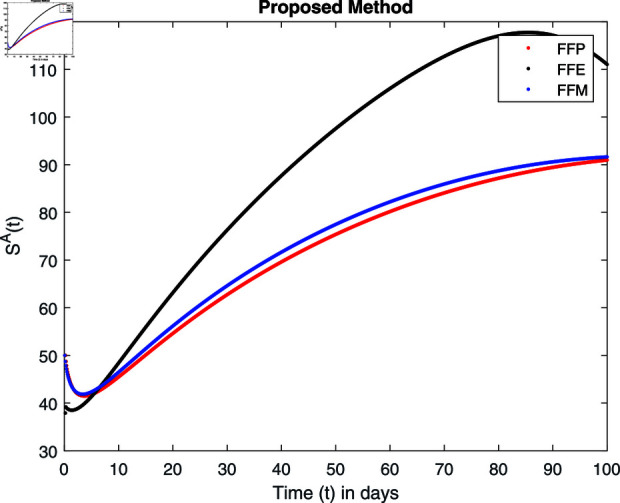
Simulation with fractional value 0.9 and fractal dimension 0.8.

**Fig 30:**
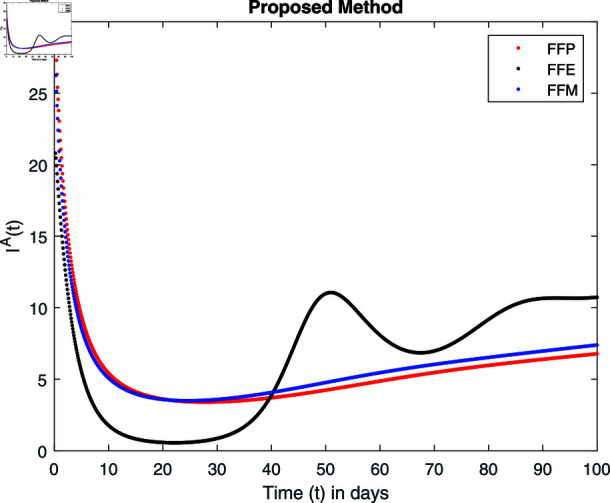
Simulation with fractional value 0.8 and fractal dimension 1.

**Fig 31:**
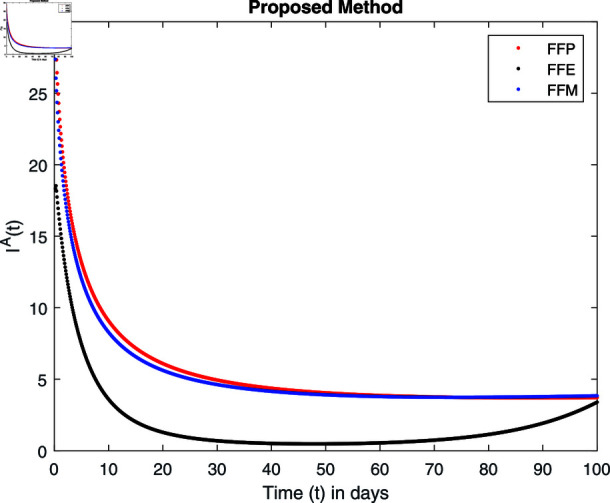
Simulation with fractional value 0.8 and fractal dimension 0.8.

**Fig 32:**
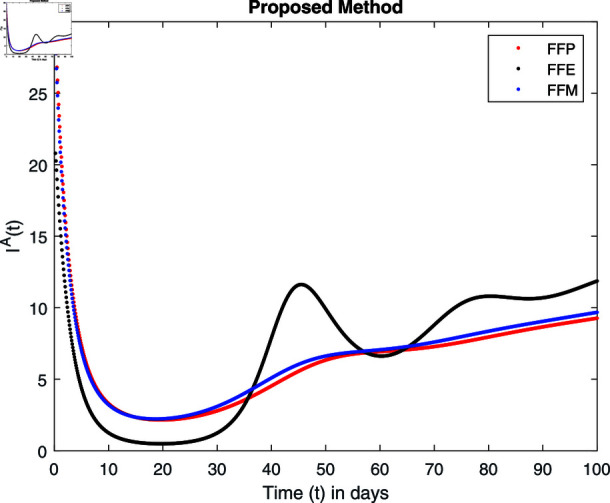
Simulation with fractional value 0.9 and fractal dimension 1.

**Fig 33:**
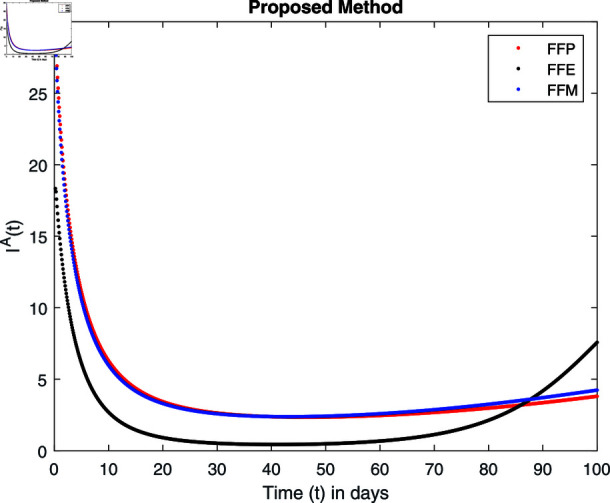
Simulation with fractional value 0.9 and fractal dimension 0.8.

**Fig 34:**
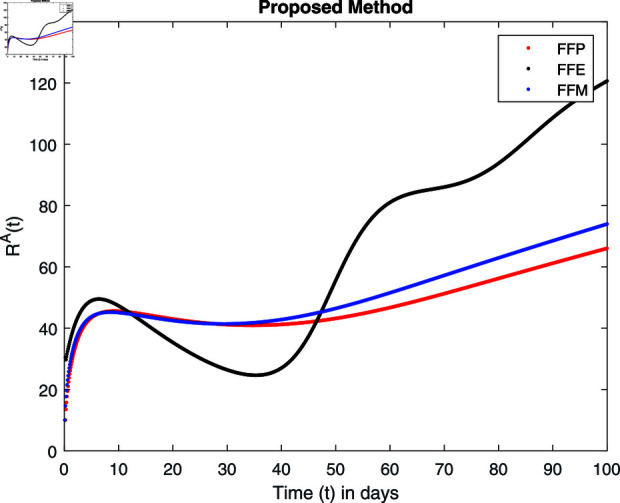
Simulation with fractional value 0.8 and fractal dimension 1.

**Fig 35:**
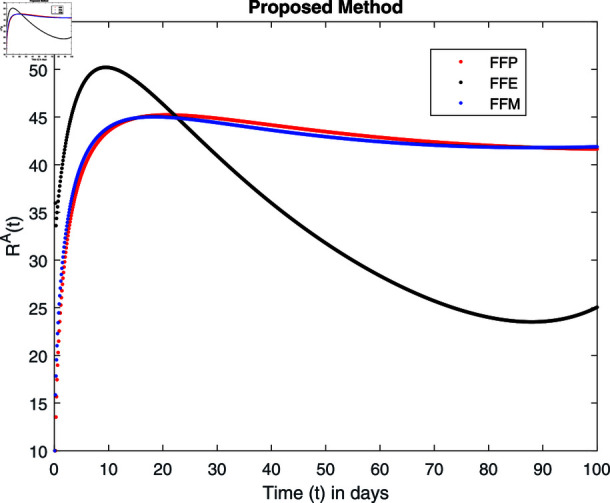
Simulation with fractional value 0.8 and fractal dimension 0.8.

**Fig 36:**
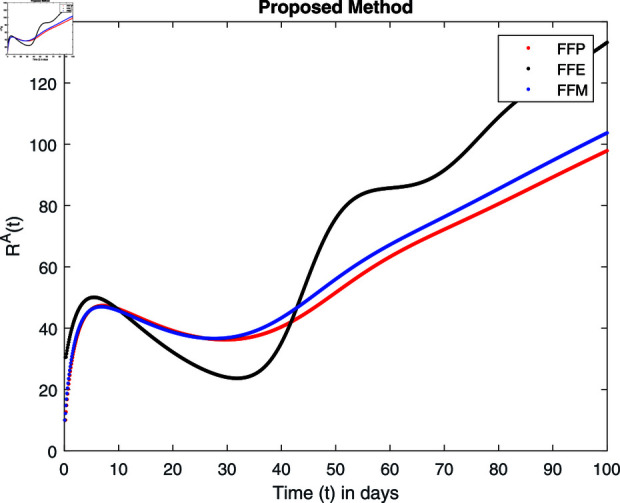
Simulation with fractional value 0.9 and fractal dimension 1.

**Fig 37:**
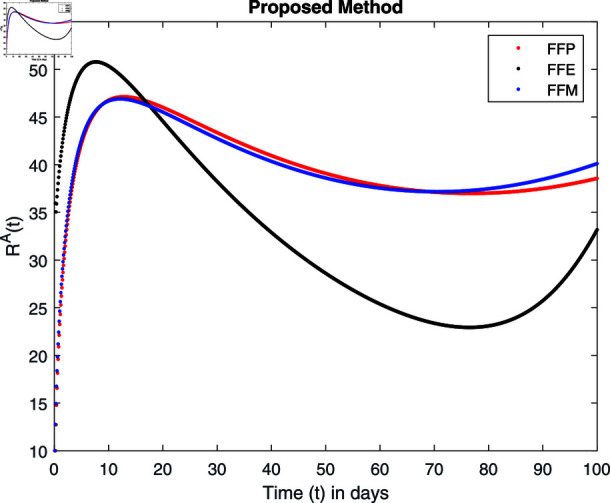
Simulation with fractional value 0.9 and fractal dimension 0.8.

## 7 Numerical simulations and discussion

Fractal fractional operator analysis, disease spread analysis, and powerful numerical techniques are used to assess the impact of our results. By means of modeling, mathematical aspects of leptospirosis transmission are examined. By varying the fractional operator’s value in relation to the surroundings, exceptional results can be achieved. The numerical simulation for various fractal and fractional dimensions of the Leptospirosis model is found using MATLAB 2018. The parameters employed are *α* = 1.6, *λ* = 0.000046, *ρ* = 0.0089, *δ* = 0.066, *ζ* = 0.0027, *ϕ* = 1.9, *Λ* = 0.0018, *η* = 0.0079, *β* = 0.05, *ω* = 0.7. The values of two parameters *ω* and *β* are assumed and the values of all other parameters are taken as studied in [49]. The initial conditions assumed are $S^{\hbar }(0)=40,I^{\hbar }(0)=20,{\mathbb{R}}^{\hbar }(0)=10,S^{A}(0)=50,I^{A}(0)=30$, and ${\mathbb{R}}^{A}(0)=10$. Figs 14, 15, 16, 17, 18, 19, 20, 21, 22, 23, 24, 25, 26, 27, 28, 29, 30, 31, 32, 33, 34, 35, 36, and 37 show the results of the simulations for two scenarios for fractional value *ξ* = 0 . 8 , 0 . 9 with *γ* = 0 . 8 and *γ* = 1 as a dimension for each sub-compartments using different kernels. Each graph clearly exhibits a unique rate of convergence based on the fractional order *ξ*; nonetheless, each curve’s distinctive feature is a steady state.

These figures show the impact of increasing or decreasing fractal and fractional values on the solution with comparison to different fractal fractional operators. One can observe that the solution of the different compartments show an abrupt change in case of FFE while the solution using FFM, FFP remain stable and are very close to each other.

Figs 14, 15, 16, and 17 show the graphical explanation of $S^{\hbar }(\tau )$ with fractional 0.8 and 0.9 and different fractal dimensions. In the case for fractional, i.e., by setting fractal value 1, the population of susceptible humans decreases after a large increase for FFE case while it increases as we decrease the fractal value to 0.8 as depicted in Figs 14, 15, 16, and 17.

Figs 18, 19, 20, and 21 show the graphical explanation of $I^{\hbar }(\tau )$ with fractional 0.8 and 0.9 and different fractal dimensions. We notice that the population of infected people shows a large increase for FFE as compared to FFP, FFM with fractal value 1, while the decrease of fractal dimension declines the population first and then approaches to its maximum value.

Figs 22, 23, 24, and 25 unnotice the behaviour of the recovered humans compartment with fractional 0.8 and 0.9 and different fractal dimensions. We can observe that the population of recovered humans first decreases and then increases to its maximum value as we choose fractional value1 0.8 and 0.9, and dimension equal to 1 or 0.8. The decrease of fractal dimension also decreases recovered populations for all three cases but this decline of population is more in FFE case as compared to FFM and FFP.

Figs 26, 27, 28, and 29 show the graphical explanation of $S^{A}(\tau )$ with fractional 0.8 and 0.9 and different fractal dimensions. In case of fractional value 0.9 and 0.8, i.e., fractional case, decline can be seen in susceptible animals populations after a large increase. This is due to the fact that animals start to be infected. As we decrease dimension value to 0.8, we see that susceptible animals increase and approach to its maximum value by varying the different dimensions.

Figs 30, 31, 32, and 33 show the graphical explanation of $I^{A}(\tau )$ with fractional 0.8 and 0.9 and different fractal dimensions. The increase in susceptible populations has a negative impact on infected population which decreases and then approaches to its equilibrium points by varying fractional and dimensional values.

Figs 34, 35, 36, and 37 show the graphical explanation of ${\mathbb{R}}^{A}(\tau )$ with fractional 0.8 and 0.9 and different fractal dimensions. One can see that there is at fractional values 0.9 and 0.8, recovered animals first decrease and then tend to their maximum value. When we decrease dimension to 0.8, then recovered population declines after a large increase.

## 8 Conclusion

We developed a mathematical of Leptospirosis disease in the context of different memories by taking combine measures of recovered human as well as animals. We provided qualitative and quantitative analysis of developed the model. First, we derived model’s disease free and endemic equilibrium points with local stability of disease free equilibrium points. After it, we find reproductive number, boundedness, positivity and bifurcation analysis. It is observed that there is no flip bifurcation occur in the newly developed system. Then we examined the Hyers Ulam stability of the model following the results on existence and uniqueness with different kernels. We used Lagrange’s interpolation polynomial- based numerical schemes, which provided us with several exciting numerical schemes, to aid in our numerical investigation. We had excellent outcomes with the plan, and it’s extremely simple to use and efficient in the study of dynamics. We gave a brief overview of our model’s simulation using novel numerical techniques. Three distinct operators allowed us to arrive at the numerical findings that were provided for the solution of the leptospirosis transmission. After choosing a few appropriate values for the fractional and fractal orders, we displayed the graphical results for each operator with making comparisons. It is evident that the model exhibits distinct behaviors for every value of the fractional order with different dimensions. Researchers and scientists that work on modeling the leptospirosis model will be able to explore new avenues with our new investigations under various operators. It can be shown from the results that fractal fractional operators, when used, yield valuable outcomes that are not visible to regular operators by reducing its dimensions. Modeling of such type will be helpful for the engineering specialties, biological scientists and other science-related domains.

This study suggests to use some natural ways to prevent leptospirosis: natural animal control techniques, safe management of trash, consume water carefully for you and your dog, avoid swimming in water that may be contaminated with Leptospirosis, treatment of dogs using Homeopathic medicines and boost the natural immune power.

Current research used theoretical and numerical approaches in the deterministic sense. In future, we aim to extend the model by comparing both determinist and stochastic models incorporating fractional technique considering different incidence rates. The same model can also be studied with other fractional derivatives, such as conformable and constant proportional caputo derivatives as discussed in [59–61]. Optimal control theory is crucial for the investigation of future outcomes of fatal diseases, which is under progress and will be stated in a future research.
